# A data-driven approach to measuring epidemiological susceptibility risk around the world

**DOI:** 10.1038/s41598-021-03322-8

**Published:** 2021-12-15

**Authors:** Alessandro Bitetto, Paola Cerchiello, Charilaos Mertzanis

**Affiliations:** 1 Department of Economics and Management, University of Pavia, Pavia, 27100 Italy; 2 College of Business, Abu Dhabi University, P.O. Box 59911, Abu Dhabi, United Arab Emirates

**Keywords:** Scientific data, Statistics, Nonlinear phenomena

## Abstract

Epidemic outbreaks are extreme events that become more frequent and severe, associated with large social and real costs. It is therefore important to assess whether countries are prepared to manage epidemiological risks. We use a fully data-driven approach to measure epidemiological susceptibility risk at the country level using time-varying information. We apply both principal component analysis (PCA) and dynamic factor model (DFM) to deal with the presence of strong cross-section dependence in the data. We conduct extensive in-sample model evaluations of 168 countries covering 17 indicators for the 2010–2019 period. The results show that the robust PCA method accounts for about 90% of total variability, whilst the DFM accounts for about 76% of the total variability. Our index could therefore provide the basis for developing risk assessments of epidemiological risk contagion. It could be also used by organizations to assess likely real consequences of epidemics with useful managerial implications.

## Introduction

During the past year, the Covid-19 pandemic has infected more than 100 million people and caused more than 2 million deaths in more than 200 countries around the world. The associated real and social costs are huge. Some estimates raise the global real cost of the Covid-19 pandemic for the next few years to several USD trillion^1^. A great concern has been the virus’ spread to countries with weaker epidemics management systems. Thus, knowing how countries with different degrees of preparedness have responded to the pandemic is important for assessing cross-country epidemiological risk and optimally deploying resources in support of this global health emergency. This is critical knowledge of globally susceptible populations, with several countries reporting infection levels exceeding their average historical levels. These policy concerns have remained valid during all phases of the Covid-19 pandemic and especially during the process of gradual adjustment of the lockdown restrictions. The question of country preparedness has surfaced again following the pandemic’s evolution^2^.

The question of countries’ preparedness to manage epidemiological risk must be addressed from a long-term perspective. It is likely that the world will continue to face epidemic risks, which many countries are still ill positioned to manage. In addition to climate change and urbanization, global population displacement and migration—now happening in nearly every corner of the world—create favorable conditions for the emergence and spread of new pathogens. Countries also face an increasing potential threat of accidental or deliberate release of deadly engineered pathogens, which could cause even greater harm than a naturally occurring pandemic. Scientific advances that help in fighting epidemic diseases have also allowed pathogens to be engineered or recreated in laboratories. Meanwhile, cross-country disparities in capacity and inattention to biological threats have exacerbated preparedness gaps. Measuring country preparedness emerges as a key real policy challenge for both countries and organizations.

We contribute to addressing this policy challenge by creating an index of epidemiological susceptibility risk (ESR) for 168 countries. Various real and non-real factors affect the extent to which a country is susceptible to epidemiological risk. We produce a new epidemiological preparedness measure that relies on objective information that facilitates policy choices. We build on previous studies and our index information accounts for the role of environmental, health, transport and communications infrastructures; real activity; demographics; and governance institutions. We deal with the complexity of these factors by implementing a fully data-driven approach to measuring their influence on epidemiological risk. In contrast to previous studies^3–5^, our fully data-driven approach produces results that provide a better evidence basis to support reasoning and decision. While there are no data-driven algorithms that can lead to fully optimal assessments of risk, our approach has considerable advantages, such as avoiding the subjective weight determination and the need for post-hoc rationalization. Evidence shows that data-driven models offer better predictive accuracy in epidemiological research than knowledge-based ones^6^. Given the complexity of the problem, we choose different versions of principal component analysis (PCA) as well as dynamic factor models (DFM) to deal with the presence of strong cross-section dependence in the data due to unobserved common factors. We conduct extensive in-sample model evaluations of 168 countries covering 17 indicators during the period 2010–2019. Our results show that the robust PCA method explains more than 90% of total variability, whilst the DFM explains about 76% of the total variability.

Our paper contributes to the literature in the following ways: it builds on previous studies by proposing a substantially improved index of epidemiological susceptibility risk that is fully model-based and data-driven, tested and validated according to advanced statistical techniques (see section Results). We use alternative analytical estimation models based on unsupervised statistical learning methods, which make neither a priori assumptions on the relationship among the input variables nor a subjective decision on the variables to be possibly dropped. Further, our data-driven approach does not need to define a target variable, thereby avoiding a further risk of subjectivity. The only model assumption lays on the number of components built on the original variable space reflecting the desired level of captured variability and predictive ability. Moreover, the new coordinates must, by construction, lie on a linear space and be orthogonal (i.e., uncorrelated). No correlation ensures that each new principal component or dynamic factor describes a specific and unknown in advance latent phenomenon through the linear combination of the initial variables. We produce the index values with different methods, which allow policy makers to assess country preparedness according to specific needs and objectives.

Moreover, our paper contributes to the multifaceted literature on the conceptualization and measurement of epidemiological risk taking a long-term perspective^7^. Indeed, most studies focus on epidemics forecasting and they do not explicitly consider the preparedness question. The key novelty of our ESR measure is the consideration of long-term, policy-relevant conditions, and not merely of the temporary incidence of diseases, affecting the contagion of epidemics. Our ESR index is not meant to predict the short-term transmission of epidemic outbreaks but rather assess the long-term risk of epidemic contagion, largely reflecting the effect of policy. Finally, our analysis complements recent risk assessments based on the use of machine learning methods^8^. Indeed, the authors stress that, beside the efficiency of the learning algorithm (often ensemble models do the job), the dataset, the selection of leading variables and the preprocessing phase in general play a key role in producing accurate assessments. We have placed special emphasis on these aspects in our analysis.

Most efforts to contain the spread and effects of epidemics use the results of prediction models^3,4,9^. The prediction of the Covid-19 behavior has deployed sophisticated methods that include big data, social media information, stochastic models and data science/machine learning techniques along with medical (symptomatic and asymptomatic) parameters^10–12^. However, prediction accuracy is limited due to the short period of data availability, data suitability, lockdown policies, difficulties in tracking the movement of people, changes in the incubation period and mutation of the virus, but also inappropriate algorithms and models.

The prediction of an epidemic establishes an alarm, which calls for a decision on what policy measures to undertake. The decision must be based on appropriate optimization of the prediction parameters, the likelihood of epidemic spread and its potential impact. Thus, it can be very complex and difficult, especially for locations with large and dense populations or critical infrastructure. Epidemics managers must factor prediction uncertainty into their decision-making models. However, while prediction methods have improved considerably and can handle increasing levels of complexity^13–15^, prediction is essentially a short-term research enterprise. Instead, the overall preparedness of a country is a crucial long-term factor that guides the making of optimal decisions in response to an epidemic prediction.

The emergence of various epidemic outbreaks in the recent years led to the formulation of various country preparedness approaches that use different information and data aggregation methods. We briefly survey the most important ones. The Global Health Security Index (GHSI) represents a comprehensive assessment and benchmarking of health security and related capabilities of the countries that participate in the WHO’s International Health Regulations. The GHSI is a joint project of the Nuclear Threat Initiative, the Johns Hopkins Center for Health Security, and The Economist Intelligence Unit^16^. The GHSI provides a measure of a country’s preparedness based on the capacity gaps of countries in their potential response to epidemics^17^. However, the GHSI has been first published in 2019 and therefore it does not provide historical data to be used in thorough real research. Further, the GHSI is too broad and includes global catastrophic and biological hazards, which on the one hand endows it with a broad coverage capacity but, on the other hand, make it less flexible and less suitable for a tool of prediction of epidemic-driven real outcomes. Najmul^18^ find insignificant correlation between the GHSI and the incidence of Covid-19. After multiple testing, they suggest the inclusion of information on demographics and the reappraisal of its aggregation methodology. Razavi et al.^19^ argue that, while very comprehensive, the GHSI scoring may not be suitable for determining priorities and comparing countries with one another, calling for a further refinement of the index process that rationalizes the index’s extensive focus on developed countries and health-related variables and its weighting methodology.

A related effort to assess country preparedness is the Joint External Evaluation (JEE) assessment tool. The latter is an externally validated, voluntary and collaborative assessment of 19 technical blocs of information necessary to validate the countries’ capacity to detect and respond to public health risks^20^. Unlike the GHSI, which allows inter-country comparisons, the JEE is a formal component of the WHO’s Monitoring and Evaluation Framework, which all UN member states must implement. The JEE is not designed for making inter-country comparisons, but instead it is a technical tool for providing support to WHO member countries in setting quantified baseline thresholds for assessing progress. Shahpar^21^ use the average of the JEE’s 19 technical areas for benchmark/comparison and argue that the JEE represents an initial effort at policy coordination that requires more global collaboration and prioritization of intervention. Garfield et al.^22^ tested the effectiveness of the JEE tool in a few African countries and found a high level of correspondence between score and policy text at the country level but also considerable differences in actual country responses relative to the benchmark JEE scores. They propose a better alignment of the JEE measures with the timing and depth of the country responses, which also reflect the contribution of international assistance in these areas.

Moreover, the Joint Research Centre (JRC), the European Commission’s science and knowledge service, has cooperated with the World Health Organization to produce the Index for Risk Management (INFORM)^23^. The latter is a composite indicator that identifies countries at risk of humanitarian crisis and disaster that would overwhelm national response capacity and would be more likely to require international assistance. The INFORM model is based on risk concepts published in scientific literature and envisages three dimensions of risk: hazards and exposure, vulnerability, and lack of coping capacity. Risk components factored into the analysis include natural disasters, socioeconomic factors, such as inequality and aid dependency, and institutional capacity, such as built environment and access to health care. However, the INFORM framework does not adequately capture the effect of biological hazards (i.e., epidemic outbreaks). The INFORM Annual meeting 2017 in Rome agreed to proceed by incorporating ancillary information from the WHO epidemiological risk initiative relating to health components to improve the overall INFORM index^24^. The index measures a wide variety of hazard risks and less so epidemiological ones and its multi-level and complex construction also makes it less flexible and less suitable for use as a policy tool.

Another comprehensive effort to develop a preparedness index was expended by the U.S. Center for Disease Control and Prevention (CDCP). Following the emergence of various national hazards, the CDCP produced the National Health Security Preparedness Index at the U.S. state level to measure the preparedness^25^. The NHSPI uses information from six broad domains of national health security^25,26^. The domains are the management of incident and information, the delivery of health-care services, the improvement of occupational and environmental health conditions, the management of countermeasures, community engagement and planning conditions, and the surveillance of health security conditions. After reviewing these occupational and environmental health domains, we observe no inclusion of indicators of occupational health and safety but only measures of environmental health. Overall, while the NHSPI is comprehensive, it covers only one country (the U.S.) for only a few years. Moreover, we do not find evidence of using the NHSPI to predict real outcomes in the US economy.

Furthermore, Marcozzi et al.^27^ present a Hospital Medical Surge Preparedness Index (HMSPI) that can be used to systematically evaluate health care facilities across the U.S. states regarding their capacity to handle patient surges during disasters. The index aims to ensure that the US health care delivery system is poised to respond to mass casualty events by assessing the ability of victims to access health care^28^ as well as resolving weaknesses and reinforcing strengths in hospital and emergency management planning and capacity^29^. The HMSPI uses four domains of surge capacity: staff, supplies, space, and integrated systems, and their subcomponents. However, the HMSPI is a static measure and of interest mainly to the US researchers.

Finally, Mertzanis et al.^5^ propose a composite index of epidemiological susceptibility risk, which they use to predict tourist flows around the world. They use information on time-varying, policy-relevant factors, such as infrastructure; demographics, real activity and institutions, which they standardize and combine based on a standard PCA method to produce a continuous value index, using equal weights. While their index proves a significant predictor of tourist flows, their methodological approach is a rather simple one depriving their index from its full predictive potential. The authors acknowledge the need for using more sophisticated dimensionality reduction methods to achieve better results. Table 1 provides a summary of key previous efforts to develop alternative composite measures of country preparedness to epidemiological risk. We acknowledge that other studies exist, mainly in epidemiological research field, that have measured aspects of epidemiological risk. However, we refer more directly to those that have had important policy implications.

**Table 1:** Comparison of alternative measures of country preparedness to epidemiological risk.

Index name	Coverage	Source
Global Health Security Index (GHSI)	Composite index, covering 195 WHO member countries, available since 2019. It measures country preparedness to respond to epidemics based on capacity gaps	The Johns Hopkins Center for Health Security & the Economist Intelligence Unit
Joint External Evaluation (JEE) Assessment Tool	Composite index, covering 195 WHO member countries, available since 2005. It measures policy gaps relative to benchmark in responding to public health risks	WHO: IHR Monitoring and Evaluation Framework
National Health Security Preparedness Index	Composite index, covering the USA only, available since 2015. It measures management efficiency in responding to public health risks	The Center for Disease Control and Prevention (CDCP)
Index for Risk Management (INFORM)	Composite index, covering 191 countries, available since 2019 (version covering epidemic risk). It measures the extent to which countries are at risk of humanitarian crisis and disaster that would overwhelm national response capacity.	Joint Research Centre (JRC), European Commission
Hospital Medical Surge Preparedness Index	Composite index, covering the USA only, available since 2015. It measures the ability of health care facilities to handle patient surges during disasters	Marcozzi et al.^27^
Epidemiological susceptibility risk index	Composite index, covering 188 countries during 2000-2019. It measures the extent to which countries are susceptible to epidemiological risk broadly accounting for health, economic and institutional factors.	Mertzanis et al.^5^

A common characteristic of the above preparedness measures is that they are composite indicators (CIs). Some indices measure preparedness using mostly health-related information, whilst others extend their coverage to include information on relevant disasters and crises, others focus on the role of environmental factors, and yet others take into consideration real and institutional factors. Thus, while structurally different, these indices capture complementary aspects of epidemiological risk manifestation. As a result, some of them may be more suitable for measuring long-term country likelihood to suffer from the outbreak of epidemics, others could better measure long-term country preparedness to respond effectively to epidemic outbreaks, whilst others may be more suitable to assess the long-term effects of epidemic outbreaks on the economy. Alternative composite measures can only capture different structural and time-relevant aspects of a phenomenon. They should therefore be properly integrated in a broader framework that considers their general and environmental repercussions^30^. Moreover, the construction involves stages where subjective judgments need to be made on the selection of indicators, the treatment of missing values, the choice of aggregation process and the weights of the indicators, etc. The unavoidable subjectivity involved in their construction may undermine their credibility and therefore it is important to identify the sources of subjectivity. However, the absence of an objective way to determine weights and the aggregation methods should not compromise their validity provided that the overall construction process is transparent^31^. This paper proposes a data-driven approach, which overcomes potential subjectivity bias in weight selection, takes into consideration dynamic effects and provides a better understanding of the complexity in approximating epidemic effects. After all, evidence-based evaluation of national epidemic management programs is critical to their future success^32^.

The conception of our ESR index originated in our observation that the spread of COVID-19 differed among countries. We observed that some countries fared better than others in containing the spread, regardless of their level of development, which was mainly the result of policy choices. The index we propose, measures country susceptibility to epidemiological risk for the 2010–2019 period based on complete annual country level data. It is worth noticing that, it may not be suitable to measure the incidence of Covid19 outbreak on a daily basis, not least because the pandemic has emerged in the last year, for which data is only partly available. Our index may be better suited to capture the impact of long-term time-varying structural factors on the contagion of epidemic outbreaks and their effect on the economy. Our index construction reflects our effort to include relevant policy variables. To this end, it reflects the importance of infrastructure, demographics, real activity and governance^5,18,19^.

The literature on epidemiological risk provides justification for these factors. First, quality health care infrastructure facilitates the timely detection and monitoring of infectious people in time and space, and therefore the successful containment of the epidemic^33^. Global coordination increases monitoring efficiency. Moreover, quality health care infrastructure helps improve productivity and employment and hence production resilience, general stability and social inclusion^34^. Adequate financing of health care infrastructure contributes decisively to its effectiveness^35^.

Second, an effective communications infrastructure improves market surveillance, raises public awareness of epidemics risks and facilitates the swift private and public responses by assembling and broadcasting suitable information^36^. A new survey finds that about 53 percent of adults in the U.S. say that the internet has been essential for dealing with the pandemic, whilst 34 percent describe it as “important, but not essential”^37^.

Third, an effective transportation infrastructure facilitates the monitoring and control of infectious population but also the response and timely provision of necessary care^38^. This is especially important with respect to passenger aviation that unavoidably contributes to the spread of an epidemic. Hufnagel et al.^39^ found a significant association between heterogeneity in airline connectivity networks and epidemic predictability.

Fourth, an effective infrastructure securing clean water and sanitation services is necessary for containing the speed and spread of epidemics and induces the health care sector’s response to adhere to high sanitary standards^40^. During epidemic outbreaks, the transmission of diseases occurs through both access to local water distribution facilities and the availability of man-made or natural water resources and sanitation systems. OECD^41^ argues that enhancing environmental health through better air quality, water and sanitation, waste management, along with efforts to safeguard biodiversity, will reduce the vulnerability of communities to the effects of epidemics. KWR^42^ found that screening for Covid-19 at municipal wastewater plants in the Netherlands contributed to a better monitoring of its spread.

Fifth, demographics is also important. The increasing life expectancy and decreasing fertility rates change the patterns of consumption thereby affecting the dynamic of epidemics. For instance, Geard et al.^43^ argue that declining fertility rates are associated with an older mean age of disease infection that affects the spread of epidemics, depending on vaccination and other policy measures. Further, the rising urbanization rate globally affects epidemics in two ways^44^: it causes improvements in health infrastructure in urban areas, but also provides a fertile ground for the emergence of new pathogens due to tighter human encounter. Population density is generally associated with a faster and wider spread of epidemics^45,46^.

Sixth, real activity also affects the spread of epidemics. Relman et al.^47^ report the views of different experts on how travel, trade and conflict move people, animals and plants globally affecting the transmission of diseases. Adda^48^ finds that booms increase people’s mobility among different transmission venues (ports, airports, etc.) and interpersonal interaction thereby contributing to a wider and faster spread of epidemics. Suhrcke et al.^49^ argue that real downturns cause higher urbanization and congestion of people seeking jobs, worsening living and health care access conditions of living, which in turn lead to adverse epidemic effects. Kafertein^50^ argued that the rapid concentration of global food trade in a few multinational corporations increased the transmission of foodborne diseases. Lang^51^ stressed the effects of mass production and logistics procedures on the spread of infectious diseases.

Finally, institutional governance matters. Quah^52^ and Pritchett et al.^53^ document from different perspectives how institutional governance, exerted through various social interactions, social coordination and risk management policies, affect the spread of epidemics. However, the capacity of governance institutions develops differently among countries, subject to political influence, uncertainty or conflict^54^. OECD^55^ argues that higher human capital improves governance and health outcomes through stronger social capital networks, employment prospects and psychological responses.

## Results

After the imputation of missing values (see Section 2 in the Supplementary Information), we standardize the dataset for each year and then we apply first the PCA method in all different versions, as described in section Results. Table 2 reports the results of the different PCA versions. We report the average variance explained by loadings across years, as well as the average $R^2$ on both the whole dataset and subsets with values trimmed for the 95th and 99th percentiles in order to check for outliers impact. In our context, in analogy with the classical $R^2$, we compute the RSS term as the squared residuals given after the reconstruction step using only the retained principal components and the TSS term as the total variance contained in the original variables. Moreover, we run the Augmented Dickey–Fuller test on the PCA index and *p* values $\ll 0.01$ for all model specifications ensure its stationarity. The stationarity is important because we can infer that the changes over time, which the index is expected to capture, can be statistically robust and not caused by any trend in the data or mean-reversion effects. The results show that the robust PCA method performed best regardless the employed data (full data set, 1% trimmed and %5 trimmed). Accordingly, we retain only the first principal component, which explains at its minimum a remarkable 87% of the total variance and therefore renders the resulting ESR index visually interpretable. Figures F3-F5 in the Supplementary Information report the scree plots of the variance explained by the loadings among all PCA methods and Figure F6 shows the relative importance of the loadings. This includes the percent of variance explained by the first principal component of each PCA method per year.

**Table 2:** Results from Robust PCA.

Method	Number of PC	Mean explained variance	Mean $R^2$	Mean $R^2$ on 99th	Mean $R^2$ on 95th	Augmented Dickey–Fuller
PCA	1	49.9 ± 0.9%	49.9 ± 0.9%	57.3 ± 1.1%	65.3 ± 0.9%	$\ll 0.01$
RobPCA	1	87 ± 0.9%	94.8 ± 0.3%	95.4 ± 0.2%	96.5 ± 0.2%	$\ll 0.01$
RobSparPCA	1	50.2 ± 0.9%	28.5 ± 3%	33.6 ± 3.6%	38.2 ± 4.5%	$\ll 0.01$

Mean is evaluated over years. Mean Explained Variance is evaluated from the eigenvalues of PCA, $R^2$ is reported for the full dataset and for the 99th and 95th percentiles. In analogy with the classical $R^2$, we compute the RSS term as the squared residuals given after the reconstruction step using only the retained principal components and the TSS term as the total variance contained in the original variables. Augmented Dickey–Fuller test for stationarity of the ESR index as well.

Then, we apply the DFM method, as described in section Results, which depends upon two hyper-parameters: the sparsity coefficient $\alpha$ of the VAR and the correlation structure of the residuals for Kalman filter. Thus, we simulate synthetic factors ${\widetilde{\mathbf{F }}}$ with different combinations of number of observed variables, countries, years, latent factors $\mathbf{F }$, and we generate the corresponding $\mathbf{y }_{t}$ given different combination of $\mathbf{A }$, defined by $\alpha$, and $\mathbf{C }$, randomly generated, using equation (1). For each combination and correlation structure of residuals $\mathbf{Q }$, we apply the described algorithm and assess the reconstruction error on the fitted factors ${\widetilde{\mathbf{F }}}$ with the simulated factors $\mathbf{F }$. The optimal parameters found are $\alpha =0.2$ and a diagonal structure. Since the Explained Variance term cannot be computed for DFM, we make use of the relative $R^2$ as defined above. Table 3 reports the DFM results. We recall that negative values of $R^2$ index can occur due to extremely poor reconstruction performance, i.e. RSS greater than TSS. In this case, the unsatisfactory performance of DFM is due to the small size of the dataset compared to the number of parameters, although mitigated with sparseness. Moreover, the estimated interactions factor in $\varvec{{\hat{A}}}$ turns out to be very small (values range in $[-0.06, 0.05]$), so we assume to be valid the no interactions setting, which has produced the highest $R^2$ (73.6%). We run the Augmented Dickey–Fuller test also on the DFM based index obtaining p-values $\ll 0.01$ for both model specifications and ensuring its stationarity as for the PCA case. Figure F7 in the Supplementary Information shows the relative importance of the loadings for the DFM model with interpretation.

**Table 3:** Results for DFM.

Method	Number of Factors	$R^2$ (%)	$R^2$ on 99th (%)	$R^2$ on 95th (%)	Augmented Dickey–Fuller
DFM with interactions	1	− 204.5	− 43.8	7.7	$\ll 0.01$
DFM without interactions	1	− 405.4	38.6	73.6	$\ll 0.01$

$R^2$ is reported for the full dataset and for the 99th and 95th percentiles. In analogy with the classical $R^2$, we compute the RSS term as the squared residuals given after the reconstruction step using only the retained principal components and the TSS term as the total variance contained in the original variables. We also report Augmented Dickey–Fuller test for stationarity of the ESR index. Negative values of $R^2$ occur because of large reconstruction error.

As robustness check, we compare the two ESR index values generated by the competing methods in terms of predictive power within a supervised analysis setting. To this end, we use the following macro-economic variables: real GDP per capita, government consumption (percent of total), price level of capital formation, trade volume, unemployment rate and outstanding loans of commercial banks. We standardize the target variables before fitting the algorithms to make the results comparable. We use both linear and non-linear data-driven learning algorithms to capture potential non-linearity effects in the data. We use alternatively the learning techniques of Random Forest, Regularized OLS (Elastic-Net), Support Vector Machine (SVM) with Radial Basis Function (RBF) kernel, Multivariate Adaptive Regression Spline (MARS) and a single layer Neural Network (NN). All the hyper-parameters are tuned with Bayesian Optimization and a 5-fold cross-validation. When fitting Elastic-Net with a single regressor, we use the OLS regression. Final performances are evaluated using a further 5-folds cross-validation and the average test set Root Mean Square Error (RMSE) is considered. The seed used to select the cross-validation folds has been kept fixed for all algorithms in order to ensure reproducible results. We provide examples of the comparison results. Table 4 (available in the Supplementary Information) shows the RMSE percent increase in predicting Unemployment rate with the single index as regressor compared to the RMSE obtained with all 17 original variables. RMSE of models which are fitted considering ESR index solely tends to increase as we would reasonably expect. However, RMSE increases are always within one standard deviation bound suggesting that a much simplified analysis based on 1 unique index is significant and largely satisfies the parsimony principle. Table 4 clearly shows that Random Forest has the lowest RMSE by employing the original 17 variables (0.079) and further the ESR index based on the DFM approach presents the minimum RMSE (0.447). Complete results for all the fitted regressions are reported in 4.1 of the Supplementary Information.

**Table 4:** RMSE in predicting Unemployment rate using continuous index as regressor.

Algorithm	RMSE index (RMSE original)	
	DFM	Robust PCA
Elastic-Net	0.999 (0.859)	0.995 (0.859)
MARS	1 (0.583)	0.924 (0.583)
Random Forest	0.447 (0.079)	0.7 (0.079)
Single Layer NN	0.994 (0.31)	0.932 (0.31)
SVM-RBF	1.024 (0.083)	0.936 (0.083)

RMSE for regression with original variables is reported in parenthesis.

**Figure 1:**
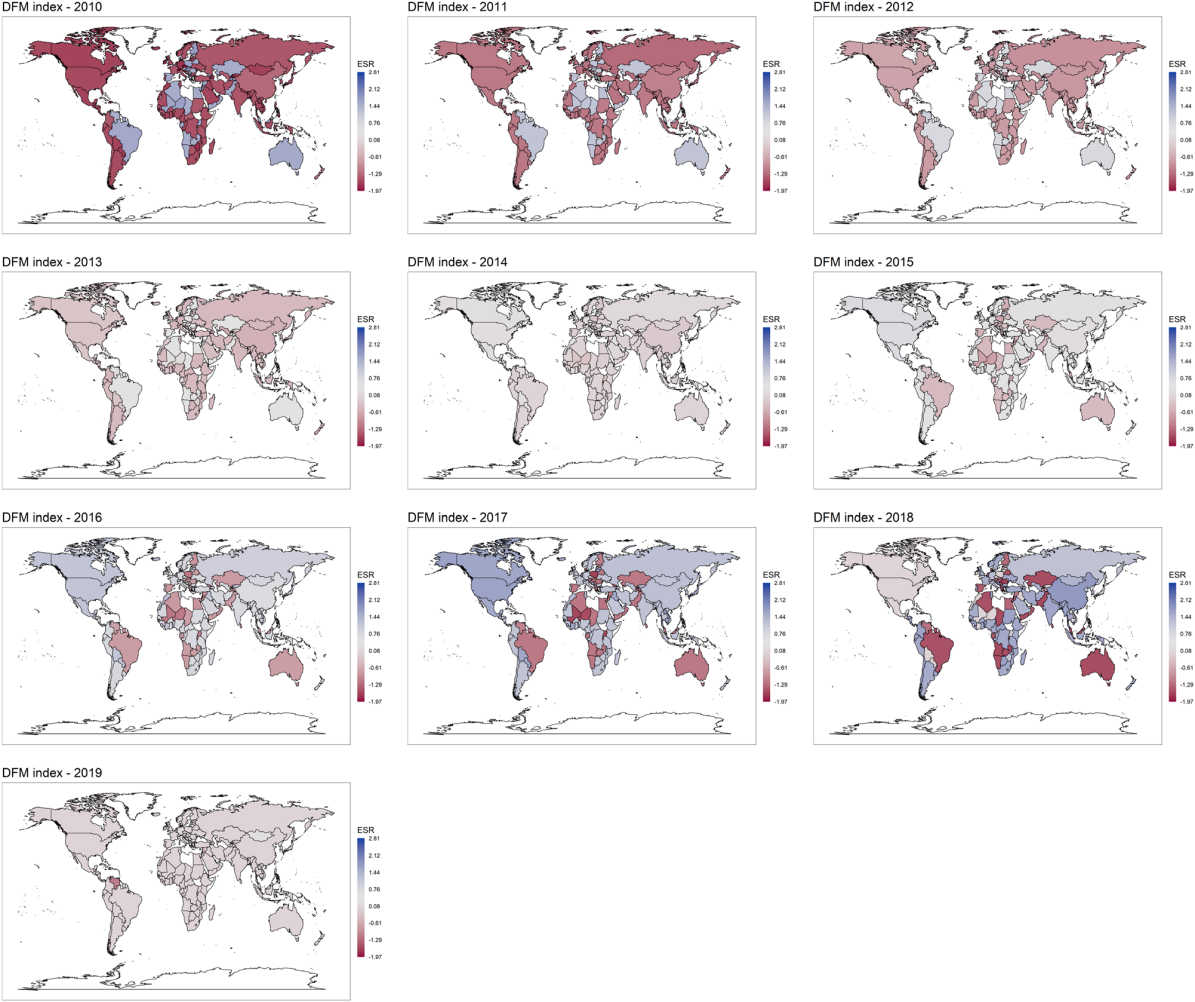
DFM index evolution over years. Shades of red color refer to riskier countries, while shades of blue to safer ones. Figure is generated with R software^56^.

Further, we can provide useful visual insights by exploring the temporal evolution of the ESR index values for each country in a world map. Figure 1 reports the global distribution of the ESR index for DFM methods (the PCA one is available in the Supplementary Information).

**Figure 2:**
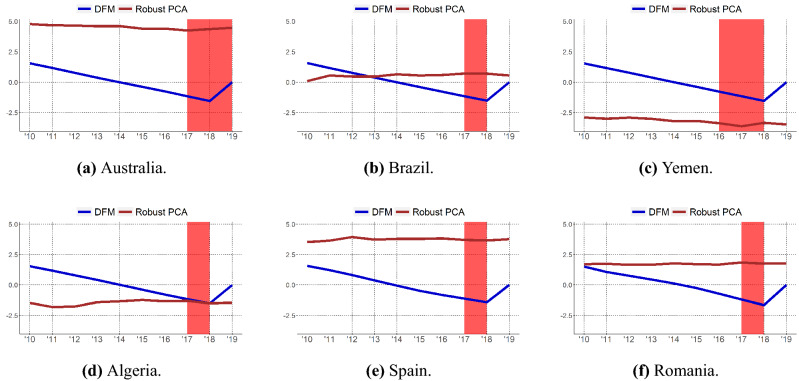
Index evolution over years for some countries. Disease outbreaks are shaded in red. Figure is generated with R software^56^.

Indeed, the native characteristic of DFM of properly modeling the temporal dynamics is reflected in the world map which presents more variability in the colour change compared to PCA. Finally, Fig. 2 shows the evolution over time of the ESR index for some individual countries, comparing the PCA and DFM methods. The PCA index is quite stable over time, whilst the DFM index captures the time dynamics of underlying latent factors. For example, Fig. 2a shows that our index can capture the abnormal increase of Influenza cases in 2018-19 in Australia. In Fig. 2b ESR index highlights the Zika virus outbreak of 2018 in Brazil. In Fig. 2c the index underlines the Cholera spread between 2016 and 2018 in Yemen. Cholera outbreak in 2018 is captured for Algeria as well as shown in Fig. 2d. Similarly Fig. 2e,f show how the index is able to capture the abnormal Influenza spread of 2018 and the increase of Measles case in 2018 in Spain and Romania respectively. Figure F14 to F17 in the Supplementary Information provide the detailed evolution of the ESR index per country during the 2010–2019 period using both PCA and DFM methods. In order to support the previous insights, we checked the Spearman correlation between our proposed ESR and the historical incidence of a number of diseases extracted from World Health Organization: HIV, Malaria, Tubercolosis (TBC) and Tropical Neglected Diseases (NTD). Table 5 reports the countries whose ESR index has the highest correlation with the corresponding disease’s evolution over the years. Only results for the DFM approach are reported. Results show the goodness of the proposed index. We can notice many high and significant correlations for all over the world countries (European, South American, African and Asian ones). The analysis suggests that the ESR index can play an important role in signaling pandemic outbreak periods thus helping regulators and countries in improving preparedness and recovery plans. Moreover, by looking at Fig. 2, we can spot the temporal evolution of both the indexes and it emerges clearly how sensitive the ESR index is to epidemic outbreaks (particularly the DFM based one).

**Table 5:** Correlation between ESR index and the historical disease incidence for HIV, Malaria, Tubercolosis (TBC) and Tropical Neglected Diseases (NTD).

Country	HIV	Malaria	TBC	NTD
Angola	1*	0.98*	1*	0.5*
Argentina	0.94*		0.67*	0.3*
Brazil	0.21*	0.43*	0.37*	0.92*
Dominican Republic	0.99*	0.32*	0.85*	0.38*
France	0.88*		0.09*	0.45
Indonesia		0.93*	0.97*	0.95*
Netherlands	0.98*		0.83*	0.28*
Nigeria	1*	0.47		0.57*
Pakistan	0.95*	0.97*	0.97*	0.1*

Only results for the DFM appoach and for the top highly correlated countries are reported.

*p val $<0.05$. Highest correlations are reported in bold.

## Discussion

Epidemic outbreaks are extreme events that are perceived by the population to be more frequent and severe, mainly due to the increased globalisation and interconnections. The COVID-19 pandemic is an extreme risk event that has unfolded with tremendous speed and breadth. Epidemics cause huge real costs for institutions and countries. It is therefore important to evaluate the extent to which countries can identify and manage epidemiological risks adequately. Despite significant improvements in infrastructure and governance worldwide, many countries remain unprepared to adequately identify and manage epidemiological risks. In this study, we have proposed a country preparedness evaluation framework that countries and institutions could use to manage the contagion and consequences of epidemic risks. The framework is based on the development of a composite indicator, which we call epidemiological susceptibility risk index (ESR), for 168 countries during 2010–2019.

In constructing our ESR measure, we use objective and regularly reproduced information that accounts for the role of infrastructure, real activity, demographics and governance institutions. This integrated view of measuring epidemiological risk is in line with the general directions proposed by the WHO. We complement previous efforts at assessing country preparedness by proposing a methodological framework that makes the assessment of preparedness more policy-driven and expanded around the world. Importantly, our proposed framework uses a data-driven approach to constructing the index that utilizes both PCA and DFM methods and their variants for achieving dimensionality reduction. The results show that, after accounting for data characteristics and missing values, the robust PCA method shows very good performance whereby the first dimension explains about 90% of total variability. However, the nature of its construction prevents it from capturing properly the temporal latent dynamic of the data. We therefore use the alternative DFM method for this purpose. Albeit somewhat less efficient in comparative terms (the first component explains about 76% of the total variability), the DFM method must be considered as the benchmark model since it properly models the temporal dynamics, which are important in capturing epidemic outbreaks across a wide range of countries during the 10 available years. Our ESR index is fully data-driven and does not allow for arbitrary and subjective choice of weights that could impair its predictive efficiency.

This framework and index could provide the basis for developing risk assessments of epidemiological risk contagion after the outbreak of an epidemic but also for ongoing monitoring of its spread and social and real effects. It would also allow for useful comparisons in country preparedness and performance. This framework and index could be used by firms to assess likely real consequences of epidemics and could therefore have managerial implications. For example, in addition to help managing epidemiological risk, the framework could be useful in aligning country and corporate policy to environmental sustainability considerations and responsible behavior. Further, it takes into consideration ongoing regulatory initiatives that stress the importance of non-financial risks due to climate change.

Finally, our framework could be revised and extended towards various directions to support decision making. One way to improve it is to increase the data series availability mindful of the missing data problem using more advanced techniques. Another way to extend it includes the addition of new relevant dimensions that may capture other aspects of epidemiological risk. As research on the sources and spread of Covid-19 continues, new information is being revealed, which might inform the re-construction of our ESR index. Another way would be to apply alternative data dimensionality reduction techniques and compare the predictive results. The extensive check on the index’s predictive power remains to be accomplished by applying it to diverse real-world situations.

## Methods

### Sources of data

The preceding literature provides the broad directions and information for constructing the epidemiological susceptibility risk index (ESR). The index broadly captures the effects of the above-described building blocks of epidemiological risk. Following previous studies, we select objective and periodically reproducible variables that, given the relevant literature, best capture the extent to which a country may be susceptible to epidemiological risk and for which there is adequate and ongoing country coverage. The index does not model restrictions per se, but the objective outcome of restrictions in terms of people and products. Our initial dataset includes the values of 17 time-varying variables for 206 countries during the 2010–2019 period, classified in seven groups to construct the ESR index: health infrastructure; environmental safety infrastructure; transport infrastructure; communications infrastructure; demographics; economic activity; and governance institutions. To capture health infrastructure effects, we use (1) the value of health expenditure per capita (current USD); (2) the index value of health care access and quality; (3) the response rate to public health hazards; (4) the number of physicians per 1,000 people; and (5) the number of hospital beds per 1,000 people. To capture transport infrastructure effects, we use (6) the (inverse of the) number of air passengers as a percent of total population. To capture demographic effects, we use (7) the number of urban populations as a percent of total population; (8) the number of people per Km2 of land (population density); and (9) the population of 65+ years of age as a percent of total population. To capture environmental safety infrastructure effects, we use (10) the number of people using safely managed drinking water services as a percent of total population; (11) the number of people using safely managed sanitation services as a percent of total population. To capture relevant real activity effects, we use (12) the value of trade in services as a percent of total trade and (13) the value of trade in goods as a percent of total trade. To capture communications infrastructure, we use (14) the number of individuals using the internet as a percent of total population. Finally, to capture institutional effectiveness, we use (15) the extent of human capital development; (16) the value of government effectiveness indicator and (17) the value of the rule of law indicator. The World Health Organization (WHO) database provides the data for variables (1) to (4); the World Development Indicators (WDI) database provides the data for variables (5) to (15); the Penn Tables (PT) database provides the data for variable (16) and the Worldwide Governance Indicators (WGI) database provides the data for variables (17) to (18). For sake of clarity, we stress that 3 out of the 17 considered variables are different in terms of measurement levels. Indeed, the human capital index (13), the value of government effectiveness indicator (16) and the value of the rule of law indicator (17) are indexes built upon other variables. However, this does not imply problems in the model specifications since they allow to take into account a wider range of information without adding more noise and keeping compact the model. A similar approach was followed by Cevik et al.^57^, Creane et al.^58^, Brave et al.^59^ and Sales et al.^60^.

Tables T1 and T2 in the Supplementary Information present the summary statistics of the index’s constituent variables Var1 to Var17 and their pairwise correlations. In order to ensure the adequate sample size suitable for the presented methodologies we run the Kaiser–Meyer–Olkin test^61^ resulting in the large score of $84.5\%$. Moreover, we run the Im-Pesaran-Shin test^62^ obtaining p-values $p\ll 0.01$ for both model specifications, i.e. “individual intercepts” and “individual intercepts and trends” for the underlying Augmented Dickey-Fuller test, implying the acceptance of alternative hypothesis of stationarity for the input variables time-series.

Higher values of these variables are associated with a lower risk of a country being susceptible to epidemiological contagion or, alternatively, they indicate better preparedness to manage these risks. While there are other relevant variables, the selected variables reflect factors and conditions that the literature has highlighted; they are objectively (not perceived) measured across countries, exhibit a low incidence of missing values and they are reproducible on a periodic basis. We did not include time-invariant factors (e.g., culture, religion, genetics) for we intend the index to capture mainly policy-relevant dynamic influences. For the same reason, we did not include time-varying factors relating to the environment conditions (e.g., temperature, rainfall) and slowly changing institutional factors (e.g., legal systems). We believe these factors should act as external controls mediating the predictive effectiveness of the ESR index on real behavior rather than being constituent elements of the index itself. We do acknowledge the limitation of choosing certain variables than others or many more, but we had to draw the line somewhere. We do believe there is room for future improvements in the index’s conceptualization and construction. An advantage of this construction is that our ESR index is mainly a policy-based and not a perceptions-based measure, which allows us to explore its effects on real behavior largely devoid of perceptions, which would make it more severely prone to endogeneity.

### Dimensionality reduction

The aim of our analysis is to extract a synthetic indicator that summarizes at best the relationship among variables in a lower dimensional space. We apply two alternative but complementary statistical methodologies to reduce dimensionality and construct the index: Principal Component Analysis (*PCA*) and Factor Analysis (*FA*). *PCA* aims at creating new variables from a larger set of observed covariates, where each one is a linear combination of the *Y* original variables. The model is represented by the equation $C=w_{1}Y_{1}+\ldots +w_{i}Y_{p}$, where *C* is the new principal component, $Y_i$ are the original variables and $w_i$ are the weights of the linear combination for $i=1,\ldots ,p$.

*FA*, on the other hand, models the measurement of latent variables, seen through the relationships they cause in a set of *Y* variables. The model is represented by a set of equations $Y_{i}=b_{i}F_{i}+u_{i}, i=1,\ldots ,p$, where $Y_i$ are the original variables, $F_i$ are the latent factors and $b_i$, $u_i$ are the parameters of the combination.

Recalling that our dataset has three dimensions, *Country*, *Variable* and *Time*, we use PCA to model country/variable interaction for each year whereas FA to model country/time interaction, for all variables. Thus, using PCA, we create a low dimensional (1 way) indicator, explaining the maximum variance of the data and considering each year separately. Whereas, using FA, we estimate a single latent component able to capture the temporal interactions among the original variables. We describe the application of each dimensionality reduction method below in more detail.

We evaluate PCA on each year separately, producing *T* models. To ensure the stability and robustness of results, we apply and compare three different PCA techniques: regular PCA, Robust PCA and Robust Sparse PCA. PCA aims at finding new and wise linear combinations of the original data, in a way that the amount of explained variance of the data is maximised. Those combinations are mathematically constrained to be mutually orthogonal (that is uncorrelated) and are called Principal Components (PC) or loadings. Given a $n\times p$ data matrix $\mathbf{X }$, where *n* is the number of observations and *p* is the number of variables, we want to find the $k\times p$ Principal Component matrix $\textit{C}$, with usually $k<<p$ such that the projected data matrix $\textit{W}=\textit{X}{} \textit{C}^T$, also called scores matrix, will have dimension $n\times k$. The maximization problem is stated as follows:

$$\begin{aligned} {\mathop {\hbox {minimize}}\limits _{\mathbf{C }}}&\quad {\Vert \mathbf{X }-\mathbf{XCC} ^T\Vert _{F}^2}\\ \hbox {Subject to}&\quad {\mathbf{C }^T\mathbf{C }=\mathbf{I }} \end{aligned}$$

where $\Vert \cdot \Vert _F$ is the Frobenius norm. We implement the model using *R*^56^ function +prcomp+^56^. Since we do not rely on the classical PCA but, rather, we seek for a robust estimation of the Principal Components, we can decompose the data matrix *X* into a low rank component *L* that represents the intrinsic low dimensional features and an outlier component *S* that captures anomalies in the data. The maximization problem is stated as follows:

$$\begin{aligned} {\mathop {\hbox {minimize}}\limits _{\mathbf{L }, \mathbf{S }}}&\quad {\Vert \mathbf{L }\Vert _*+ \lambda \Vert \mathbf{S }\Vert _1}\\ \hbox {Subject to}&\quad {\mathbf{L }+\mathbf{S }=\mathbf{X }} \end{aligned}$$

where $\Vert L\Vert _{*}$ is the nuclear norm and $\lambda$ is a penalization term. Following the procedure of Candes et al.^63^, once fitted, $\mathbf{L }$ can be used as a proxy for **X** with the extreme values excluded. Finally, following Erichson et al.^64^, we produce both a robust estimation and a sparse representation of the principal components by adding a sparsity constraint on the matrix *C*. The associated maximization problem is stated as follows:

$$\begin{aligned} {\mathop {\hbox {minimize}}\limits _{\mathbf{C }, \mathbf{W }}}&\quad {\Vert \mathbf{X }-\mathbf{WC} ^T-\mathbf{S }\Vert _F^2 + \psi (\mathbf{C }) + \phi (\mathbf{W }) + \lambda \Vert \mathbf{S }\Vert _1} \\ \hbox {Subject to}&\quad {\mathbf{C }^T\mathbf{C }=\mathbf{I }} \end{aligned}$$

$\psi$ and $\phi$ are regularizing functions (i.e. LASSO or Elastic Net).

### Dynamic factor model

Moreover, we evaluate a temporal dependent version of FA called Dynamic Factor Model (DFM), using all the available years within the same model. Given the $p\times n$ matrix $\mathbf{X }$, the model assumes that there exist some $k\times n$ factors $\mathbf{F }$ such that their mutual interaction over time can be expressed by a $k\times k$ interaction matrix $\mathbf{A }$ and the observed variable can be expressed as a linear function of the factors themselves through a $p\times k$ loading matrix $\mathbf{C }$. The problem can be solved as a system of equations:

1 $$\begin{aligned} {\left\{ \begin{array}{ll} \mathbf{F }_{t}=\mathbf{A }\mathbf{F }_{t-1}+{\mathcal {N}}(0,\mathbf{Q })\\ \mathbf{X }_{t}=\mathbf{C }\mathbf{F }_{t}+{\mathcal {N}}(0,\mathbf{R }) \end{array}\right. } \end{aligned}$$

where ${\mathcal {N}}$ is the normal probability distribution and $\mathbf{Q }$ and $\mathbf{R }$ are the covariance matrix of the residuals of each equation in (1), respectively. Due to the short time series of the input variables, this model cannot be fitted considering all countries together as the resulting system of equations (1) is under-determined. Thus, we deal with the problem as follows: first, following Holmes et al.^65^, we fit DFM for each country, obtaining the factor matrices $\mathbf{F }^i$, the factor interactions $\mathbf{A }^i$ and the factor loadings $\mathbf{C }^i$, $i=1,\ldots ,n$. Second, we fit a Vector Auto Regressive (VAR) model in order to get $\varvec{{\hat{A}}}$ 1-year lag matrix that incorporates cross-countries interactions of $\mathbf{A }^i$. We implement the model using *R*^56^ package +sparsevar+^66^ because this calibration problem has too many parameters to estimate relative to the number of observations, thus requiring a sparse approach. Finally, we use Kalman Filter to get smoothed factors $\widehat{\mathbf{F }^i}$ using $\varvec{{\hat{A}}}$ and $\varvec{{\hat{C}}}=diag(\varvec{C}^i)$, that is to get latent factors that incorporate cross-countries interactions. Briefly, Kalman filter re-estimates the factor matrix $\mathbf{F }$ iterating the two equations in (1) until the error between the predicted observed variables $\varvec{{\hat{X}}}$ and the true one is minimized. We implement the model using *R*^56^ package +FKF+^67^. We assume $\varvec{{\hat{C}}}$ to be diagonal in order not to double-count correlations within the observed variables and because cross-country interactions are already modelled through the VAR.

In both cases (PCA and DFM), the final index ESR will be represented by the scores matrix *W* and the factor matrix *F* respectively, both *k*-dimensional. One of the goal is to select the optimal number of components *k* as a trade-off between the maximal explained variance and the smallest value of components *k*.

### Validation

Applying a dimensionality reduction technique by merely maximising the amount of explained variance with the smallest set of components, could be misleading and conduct to hardly interpretable results. Thus, once identified the most reliable results, we compare the fitting power of the produced indexes to a baseline benchmark. We accordingly estimate several parametric and non-parametric regression models to produce comparisons of the produced ESR index with the original set of variables. We use, as target variable, the following macro-economic variables: real GDP per capita, government consumption (percent of total), price level of capital formation, trade volume, unemployment rate, outstanding loans of commercial banks. Our validation process aims at demonstrating the relevance of the new index in representing the information conveyed by the original component variables. If the modeling ability of the composite ESR index, measured by the root mean square error (RMSE), is comparable to the original one based on the initial variables, we can conclude that the produced indicator is not only satisfactory according to the chosen dimension reduction technique but also effective in terms of predictive power within a simplified framework.

## Supplementary Information

Supplementary Information.

## Data Availability

The World Health Organization (WHO) data can be found at https://www.who.int/data/collections; the World Development Indicators (WDI) data can be found at https://databank.worldbank.org/source/world-development-indicators; the Penn Tables (PT) data can be found at https://www.rug.nl/ggdc/productivity/pwt/?lang=en and the Worldwide Governance Indicators (WGI) data can be found at https://databank.worldbank.org/source/worldwide-governance-indicators.
