# Supplementary material for: A data-driven approach to measuring epidemiological susceptibility risk around the world
Source: Sci Rep. 2021 Dec 15;11:24037. doi: 10.1038/s41598-021-03322-8 (PMC8674252; doi:10.1038/s41598-021-03322-8)
Supplement: Supplementary file 1 — Supplementary Information. [file 41598_2021_3322_MOESM1_ESM.pdf]

Supplementary information

A DATA-DRIVEN APPROACH TO MEASURING EPIDEMIOLOGICAL SUSCEPTIBILITY RISK AROUND THE WORLD

Bitetto A., Cerchiello P., Mertzanis C.

1 List of variables and countries

Supplementary Table T1: List of used variable. Sources are World Health Organization (WHO), World Bank's Development Indicators (WDI), Penn Tables (PT) and World Bank's Worldwide Governance Indicators (WGI).

Variable	Description	Source	Total Obs.	Missing Values	Min	Max	Mean	Median	Standard Deviation
var1	health care exenditure per capita	WHO	1,680	523 (31%)	12.64	10,014.71	1,077.66	317.86	1,821.28
var2	health care access and quality	WHO	1,680	20 (1.2%)	28.60	93.60	62.97	62.55	16.49
var3	response level (%) to public health hazards	WHO	1,680	670 (40%)	0.00	100.00	66.15	73.00	30.61
var4	num of physicians per 1000 people	WDI	1,680	941 (56%)	0.00	6.11	2.01	2.05	1.42
var5	num of hospital beds per 1000 people	WDI	1,680	1175 (70%)	0.10	13.40	3.25	2.70	2.31
var6	num of air passengers to population ratio	WDI	1,680	397 (24%)	0.00	34.53	1.18	0.29	2.87
var7	num of urban pop (% of total)	WDI	1,680	168 (10%)	10.64	100.00	58.91	59.48	22.19
var8	num of people per Km2 (pop density)	WDI	1,680	177 (11%)	1.75	7,953.00	231.38	81.13	808.73
var9	num of people age 65% (% of total)	WDI	1,680	177 (11%)	0.69	27.58	8.38	6.20	5.93
var10	num of people using drinking water services (% of pop)	WDI	1,680	340 (20%)	33.05	100.00	86.51	94.72	16.70
var11	num of people using safely-managed drinking water services (% of pop)	WDI	1,680	952 (57%)	6.19	100.00	76.98	91.52	26.98
var12	num of people using safely-managed sanitation services (% of pop)	WDI	1,680	1024 (61%)	7.45	100.00	66.76	76.01	28.97
var13	human capital index	WDI	1,680	218 (13%)	0.00	4.01	2.51	2.64	0.84
var14	num of people using the internet (% of pop)	WDI	1,680	257 (15%)	0.25	100.00	45.73	45.96	29.14
var15	value of trade (% GDP)	PT	1,680	103 (6.1%)	0.20	442.62	91.30	79.51	58.12
var16	government effectiveness index	WGI	1,680	20 (1.2%)	-2.28	2.24	0.02	-0.08	0.97
var17	rule of law index	WGI	1,680	20 (1.2%)	-2.32	2.10	-0.03	-0.24	0.98

Supplementary Table T2: Correlation matrix of input variables.
var1 is health care exenditure per capita, var2 is health care access and quality, var3 is response level (%) to public health hazards, var4 is num of physicians per 1000 people, var5 is num of hospital beds per 1000 people, var6 is num of air passengers to population ratio, var7 is num of urban pop (% of total), var8 is num of people per Km2 (pop density), var9 is num of people age 65% (% of total), var10 is num of people using drinking water services (% of pop), var11 is num of people using safely-managed drinking water services (% of pop), var12 is num of people using safely-managed sanitation services (% of pop), var13 is human capital index, var14 is num of people using the internet (% of pop), var15 is value of trade (% GDP), var16 is government effectiveness index, var17 is rule of law index.

	var1	var2	var3	var4	var5	var6	var7	var8	var9	var10	var11	var12	var13	var14	var15	var16
var2	0.66*															
var3	0.35*	0.5*														
var4	0.57*	0.75*	0.44*													
var5	0.32*	0.52*	0.27*	0.64*												
var6	0.33*	0.32*	0.17*	0.21*	0.01											
var7	0.48*	0.7*	0.39*	0.58*	0.31*	0.22*										
var8	-0.04	0.15*	0.13*	-0.04	-0.02	0.2*	0.19*									
var9	0.61*	0.79*	0.38*	0.76*	0.67*	0.13*	0.46*	0.07*								
var10	0.41*	0.79*	0.42*	0.65*	0.37*	0.22*	0.64*	0.12*	0.61*							
var11	0.43*	0.53*	0.22*	0.5*	0.31*	0.26*	0.5*	0.14*	0.49*	0.36*						
var12	0.38*	0.29*	0.22*	0.27*	0.28*	0.16*	0.29*	0.14*	0.27*	-0.09*	0.72*					
var13	0.53*	0.67*	0.43*	0.54*	0.46*	0.16*	0.5*	0.12*	0.62*	0.57*	0.35*	0.34*				
var14	0.63*	0.86*	0.51*	0.66*	0.48*	0.36*	0.69*	0.15*	0.69*	0.73*	0.53*	0.31*	0.6*			
var15	0.12*	0.31*	0.06*	0.16*	0.19*	0.32*	0.29*	0.55*	0.23*	0.26*	0.2*	0.14*	0.19*	0.32*		
var16	0.71*	0.81*	0.47*	0.6*	0.39*	0.36*	0.58*	0.24*	0.72*	0.66*	0.4*	0.28*	0.63*	0.79*	0.37*	
var17	0.73*	0.76*	0.42*	0.57*	0.37*	0.38*	0.53*	0.22*	0.69*	0.58*	0.41*	0.32*	0.56*	0.75*	0.37*	0.95*

$*$ p-val < 0.05

Supplementary Table T3: Complete list of selected countries and relative missing values count and percentage over total number of observations.

Country	Missing Values	Country	Missing Values	Country	Missing Values
Nigeria	91 (6.7%)	Philippines	276 (20.3%)	Slovak Republic	336 (24.7%)
Sri Lanka	92 (6.8%)	Costa Rica	277 (20.4%)	Latvia	337 (24.8%)
Armenia	103 (7.6%)	St. Vincent and the Grenadines	278 (20.4%)	Serbia	337 (24.8%)
Lao PDR	105 (7.7%)	Mali	280 (20.6%)	Spain	337 (24.8%)
Mongolia	106 (7.8%)	Yemen, Rep.	280 (20.6%)	Austria	341 (25.1%)
Bolivia	113 (8.3%)	Guinea-Bissau	283 (20.8%)	Trinidad and Tobago	342 (25.1%)
Honduras	117 (8.6%)	China	285 (21%)	Belgium	347 (25.5%)
Moldova	122 (9%)	Indonesia	285 (21%)	Tunisia	347 (25.5%)
Nicaragua	123 (9%)	Liberia	286 (21%)	Eswatini	348 (25.6%)
Sierra Leone	129 (9.5%)	Croatia	288 (21.2%)	Romania	350 (25.7%)
Tanzania	130 (9.6%)	Ecuador	289 (21.2%)	Qatar	354 (26%)
Mauritania	134 (9.9%)	Malaysia	288 (21.2%)	Mauritius	355 (26.1%)
Benin	138 (10.1%)	Chile	292 (21.5%)	Kazakhstan	357 (26.2%)
India	139 (10.2%)	Hungary	292 (21.5%)	Bulgaria	359 (26.4%)
Kenya	142 (10.4%)	Singapore	293 (21.5%)	Malta	359 (26.4%)
Togo	141 (10.4%)	Djibouti	296 (21.8%)	Fiji	362 (26.6%)
Cote d'Ivoire	146 (10.7%)	Malawi	298 (21.9%)	Turkey	362 (26.6%)
Cameroon	150 (11%)	Sweden	300 (22.1%)	Luxembourg	363 (26.7%)
Burundi	151 (11.1%)	Peru	302 (22.2%)	Uruguay	365 (26.8%)
Mozambique	151 (11.1%)	Egypt, Arab Rep.	303 (22.3%)	Ukraine	367 (27%)
Tajikistan	152 (11.2%)	Brazil	305 (22.4%)	Finland	369 (27.1%)
Georgia	159 (11.7%)	South Africa	305 (22.4%)	Botswana	373 (27.4%)
Burkina Faso	163 (12%)	Thailand	304 (22.4%)	Denmark	372 (27.4%)
Niger	163 (12%)	Iran, Islamic Rep.	310 (22.8%)	Lebanon	372 (27.4%)
Bangladesh	165 (12.1%)	Switzerland	310 (22.8%)	Israel	375 (27.6%)
Angola	169 (12.4%)	Dominica	311 (22.9%)	Oman	376 (27.6%)
Rwanda	169 (12.4%)	Canada	313 (23%)	Portugal	376 (27.6%)
Zimbabwe	168 (12.4%)	Lithuania	313 (23%)	Norway	377 (27.7%)
Sudan	170 (12.5%)	Argentina	316 (23.2%)	Saudi Arabia	379 (27.9%)
Vietnam	170 (12.5%)	Jordan	315 (23.2%)	Germany	381 (28%)
Senegal	175 (12.9%)	Uzbekistan	315 (23.2%)	Iceland	382 (28.1%)
Bosnia and Herzegovina	179 (13.2%)	Australia	317 (23.3%)	Algeria	386 (28.4%)
Central African Republic	185 (13.6%)	Czech Republic	317 (23.3%)	Ireland	386 (28.4%)
Lesotho	185 (13.6%)	Guatemala	317 (23.3%)	Namibia	391 (28.7%)
Cambodia	194 (14.3%)	Jamaica	318 (23.4%)	Suriname	390 (28.7%)
Ethiopia	197 (14.5%)	Japan	318 (23.4%)	United Kingdom	392 (28.8%)
Sao Tome and Principe	198 (14.6%)	Dominican Republic	320 (23.5%)	North Macedonia	396 (29.1%)
Kyrgyz Republic	200 (14.7%)	Iraq	320 (23.5%)	Cyprus	399 (29.3%)
Pakistan	201 (14.8%)	Morocco	319 (23.5%)	Italy	403 (29.6%)
Bhutan	210 (15.4%)	France	322 (23.7%)	Gabon	410 (30.1%)
Nepal	209 (15.4%)	New Zealand	322 (23.7%)	Azerbaijan	411 (30.2%)
Ghana	214 (15.7%)	Panama	322 (23.7%)	Belarus	425 (31.2%)
Guinea	223 (16.4%)	Estonia	323 (23.8%)	Greece	430 (31.6%)
Uganda	223 (16.4%)	Grenada	325 (23.9%)	El Salvador	437 (32.1%)
Zambia	230 (16.9%)	United States	325 (23.9%)	United Arab Emirates	439 (32.3%)
Cabo Verde	232 (17.1%)	Kuwait	326 (24%)	Bahamas, The	444 (32.6%)
Chad	236 (17.4%)	Netherlands	327 (24%)	Belize	455 (33.5%)
Myanmar	237 (17.4%)	Russian Federation	326 (24%)	Brunei Darussalam	464 (34.1%)
Congo, Rep.	244 (17.9%)	Venezuela, RB	327 (24%)	Seychelles	477 (35.1%)
Gambia, The	243 (17.9%)	Paraguay	329 (24.2%)	Hong Kong SAR, China	482 (35.4%)
Madagascar	250 (18.4%)	Poland	330 (24.3%)	Montenegro	483 (35.5%)
Haiti	254 (18.7%)	Korea, Rep.	333 (24.5%)	Antigua and Barbuda	492 (36.2%)
St. Lucia	265 (19.5%)	Comoros	335 (24.6%)	Albania	507 (37.3%)
Congo, Dem. Rep.	268 (19.7%)	Slovenia	335 (24.6%)	Equatorial Guinea	523 (38.5%)
Mexico	274 (20.1%)	Bahrain	336 (24.7%)	Syrian Arab Republic	529 (38.9%)
Colombia	276 (20.3%)	Barbados	336 (24.7%)	Afghanistan	535 (39.3%)

In table T4 we report the distribution over time of the missing values quota, as to evaluate the impact of missing data imputation. It clearly emerges the highest quota for the last two available years.

In Figure F1 we report the evolution of the ERS index over years based on PCA.

Supplementary Table T4: Missing values over years.

Year	Total Observations	Missing Values
2010	22,848	4,367 (19.1%)
2011	22,848	3,959 (17.3%)
2012	22,848	4,273 (18.7%)
2013	22,848	4,072 (17.8%)
2014	22,848	4,019 (17.6%)
2015	22,848	4,494 (19.7%)
2016	22,848	4,404 (19.3%)
2017	22,848	4,245 (18.6%)
2018	22,848	7,478 (32.7%)
2019	22,848	8,218 (36.0%)

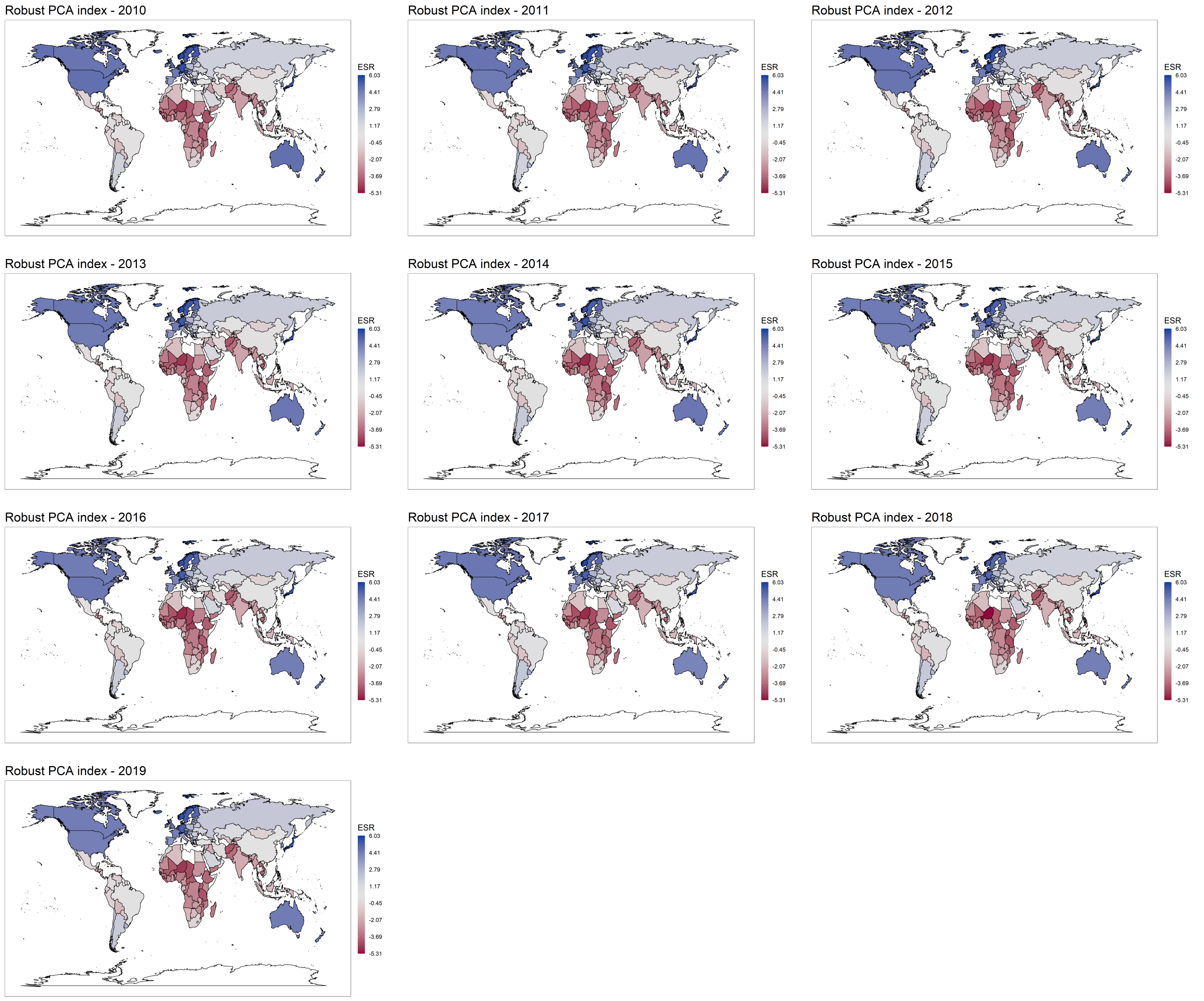

Supplementary Figure F1: Robust PCA index evolution over years. Shades of red color refer to riskier countries, while shades of blue to safer ones. Figure is generated with R software[1].

2 Missing values imputation methodology and pre-processing

Imputation of missing data

We next assess the data quality and completeness and address the problem of missing data. There are various imputation methods that are suitable for different data sets and conditions[2]. There is a trade-off between data availability and the construction of a comprehensive composite index. We stress that the goal of our index construction is not to create artificial data series. We do not use the individual series as standalone predictors. Instead, we combine them to produce a single composite index that reflects consistently epidemiological susceptibility risk, rather than data availability. King et al.[3] argue that imputation of missing data and their combination into aggregate indices is highly common in social sciences research because the nature of measured phenomena is associated with incomplete records. Typically, more data is available for larger countries and in recent years.We therefore restricted our sample to the 2010-2019 period and 168 countries (81.5% of all countries) in which the missing data tolerance rate does not exceed 40%, which gives a total of 28,560 country-year observations. Table T3 presents the full list of the sample countries and their rate of missing data, whilst Table T4 presents the missing data incidence by year.

Since the presence of many missing values can extremely impact the quality and the reliability of results, we set an operational protocol of missing values treatment and imputation. In our final sample, 114 out of 168 countries show a rate of missing data between 20-39%. To address the missing values problem that would make possible the application of robust data aggregation methods, we test two different data imputation techniques: Matrix Completion with Low Rank SVD (MC-SVD) proposed by Hastie et al.[4] and Bayesian Tensor Factorization (BTF) proposed by Khan et al.[5].

Briefly, MC-SVD solves the minimization problem $\frac{1}{2}\|X - AB^T\|_F^2 + \frac{\lambda}{2}(\|A\|_F^2 + \|B\|_F^2)$ for A and B where $\|\cdot\|_F$ is the Frobenius norm by setting to 0 the missing values. Once estimated, AB^T can approximate the original matrix X, including the missing values. This is applied on the 2-dimensional "slice" of countries-variables for each year. Subsequently, we apply the BTF method, which in addition uses a tensorial decomposition of the 3-dimensional tensors that stack all the annual “slices” together so that the imputation process involves information coming from a temporal dimension as well.

To assess imputation performances and to choose the best method, we test the algorithm in three settings. In the first setting (named *Original*) we consider the whole dataset made of 168 countries and the 17 constituents variables over 10 years for a total of 28,560 entries. The full sample has 25% of missing values, thus we randomly remove some additional values, representing 10%, 20% and 30% of the initial dataset. In the second setting (named *No missing*) we drop all entries with missing values and apply the same incremental sampling procedure on the remaining subset. In the last setting (named *Some missing*) we drop all countries with at least 3 missing values for any year and apply again the incremental sampling procedure on the remaining subset. Furthermore, we fit the two methods, MC-SVD and BTF, on the previous 3 cases with different sampling percentages and we evaluate the Mean Absolute Reconstruction Error (MARE) on the excluded observations as follows:

$$MARE = \frac{1}{M}\sum_{i}^{M}|x_{excluded} - x_{reconstructed}|$$

where M is the total number of excluded values. Moreover, we check the sensitivity to the original percentage of missing values by comparing the MARE on *No missing* and *Some missing* with the one on *Original*. Figure F2 shows bar plot of MARE values for all settings for each increasing percentage of added missing values. Bar whiskers are scaled value of $max(MARE)$, defined as:

$$RM = \frac{max(MARE)}{\text{Average value of Original matrix}}$$

In order to grasp the magnitude of the impact of MARE we also report its ratio R with the average value of the non-missing entries of original matrix:

$$R = \frac{MARE}{\text{Average value of Original matrix}}$$

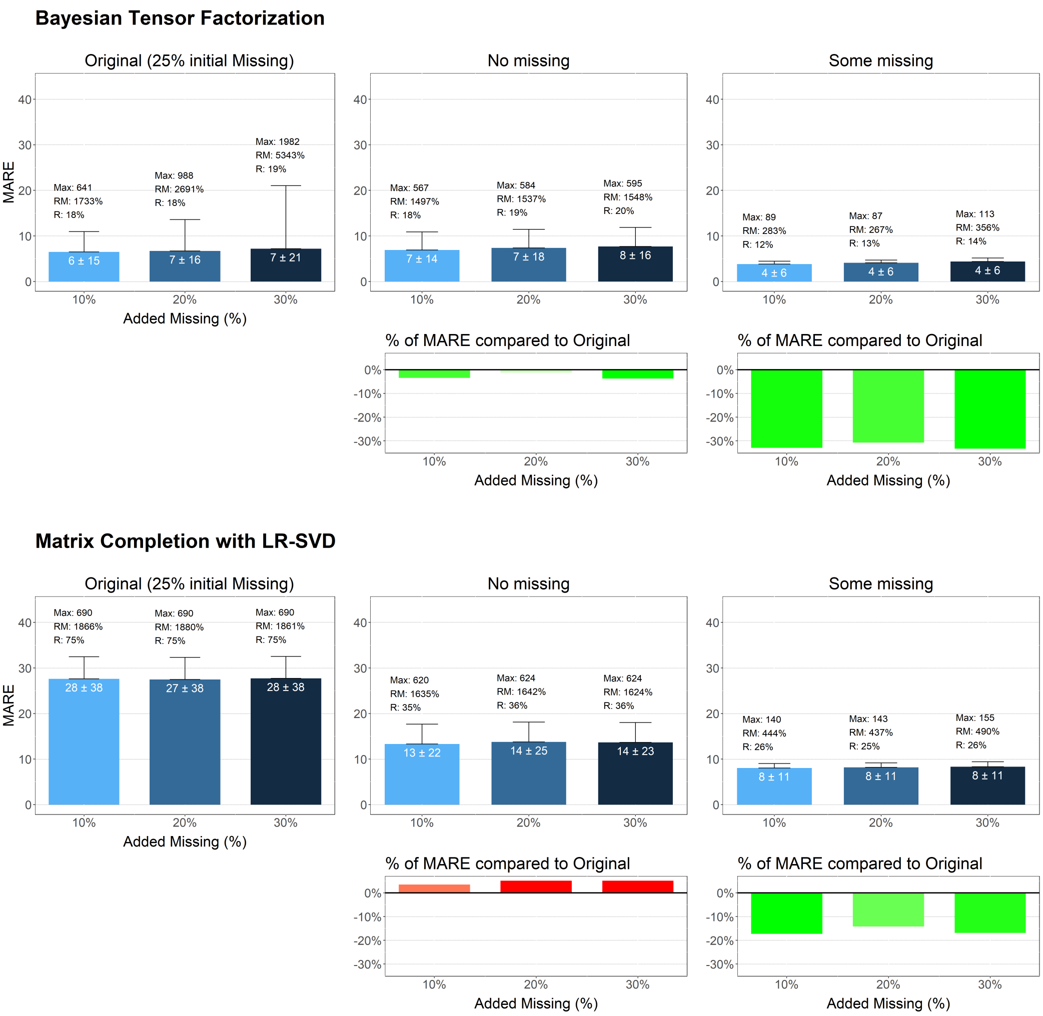

Supplementary Figure F2: Testing missing values imputation methodologies. Blue bars report the Mean Absolute Reconstruction Error (MARE), green/red bars report the percent decrease/increase of MARE compared to the one evaluated on the *Original* setting. Figure is generated with R software[1].

Finally, for the *No missing* and *Some missing* setting we show the green/red bar plot reporting the percent decrease/increase, respectively, of MARE compared to the one evaluated in the *Original* setting so to evaluate the impact of missing data in the matching entries subset. BTF has lower MARE and higher percent decrease compared to MC-SVD implying a better data reconstruction ability and reliability.

Normalization of data

We remove differences in magnitude among the input variables by standardising the values, i.e. we subtract the mean and divide by the standard deviation. Having all variables on the same reference scale is crucial for unbiased estimation when applying dimensionality reduction techniques. Standardisation relates country performance of a variable as a bounded (by unitary standard deviation) variation from an average value (set to zero by definition) across all countries and years, which facilitates variable aggregation expressed in different measurement units. Further, when applying dimensionality reduction methods, component weights can have a significant effect on the overall composite indicator and country rankings. Several weighting techniques exist[6]. Some are based on statistical models (e.g., factor analysis), whilst others are based on participatory methods (e.g., analytical hierarchy process). Regardless of the method used, weights are essentially value judgments. However, our data-driven approach overcomes the problem of arbitrary and subjective choice of weights that could constrain the index's predictive efficacy.

3 Scree Plot and Loadings Plot for PCA method

In this Supplementary Information we report scree plots and loadings of all the competing PCA approaches: Original PCA, Robust PCA, Robust Sparse PCA. If we pay attention to loadings results available in F6, we can notice that

Original PCA and Robust PCA are very similar to each other, while Robust Sparse PCA appears different for several variables (namely var2, var3, var6, var8, var12, var13, var14) because by construction it aims to a sparse and parsimonious representation. In the Robust PCA almost all the variables have a meaningful positive contribution to the first Principal Component, that constitutes our ESR index (var8 (num of people per Km2) and var15 (value of trade as % of GDP) appear to be less significant).

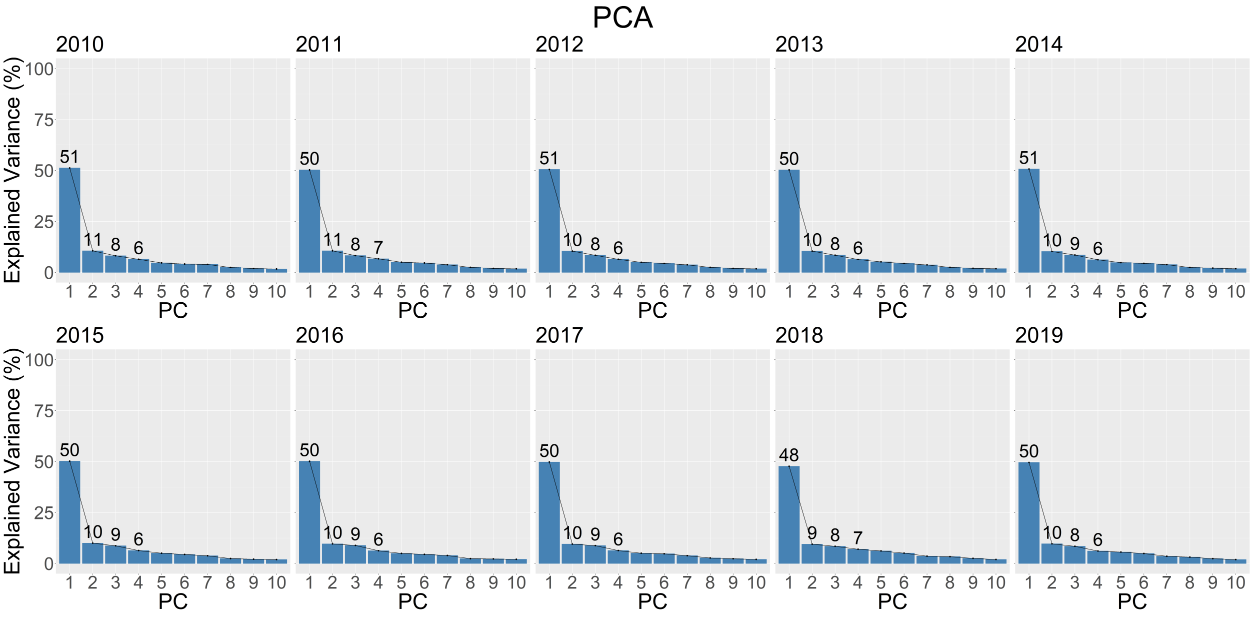

Supplementary Figure F3: Scree plot for PCA method. Figure is generated with R software[1].

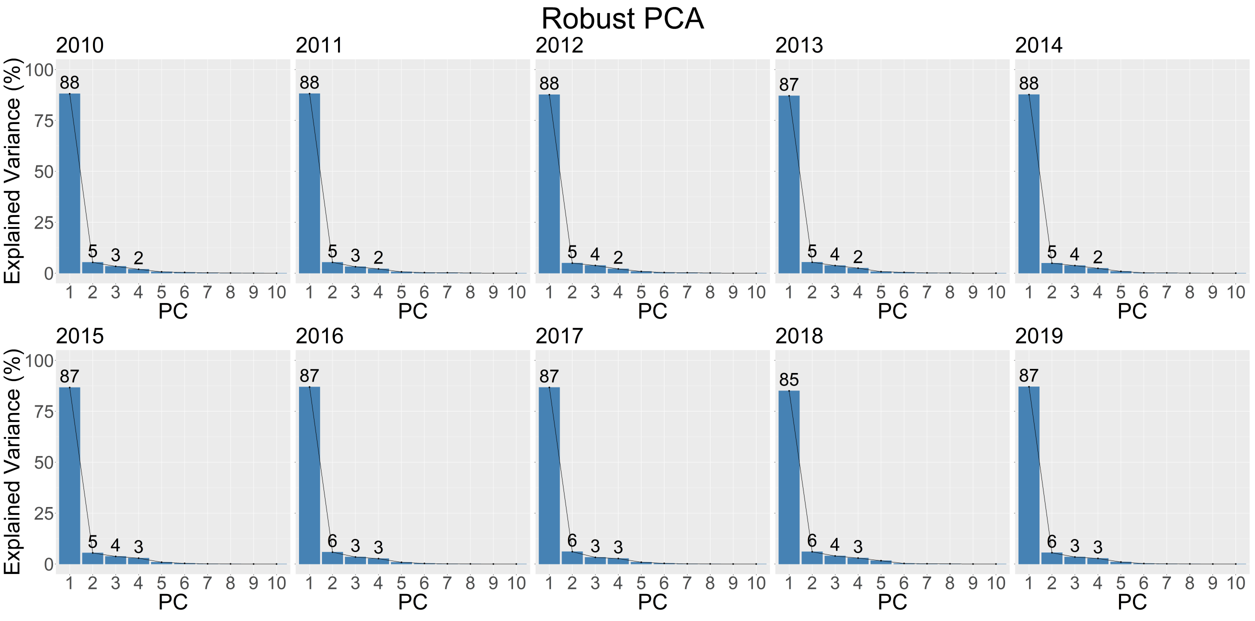

Supplementary Figure F4: Scree plot for Robust PCA method. Figure is generated with R software[1].

Figure F3 clearly shows how important is the first component whatever year we take into account. Such result has several important implications: PCA proves that there exists a strong latent component which is highly connected to almost all the variables. Moreover, the possibility of building up our ESR index considering just one component eases the interpretation, the relative employment and the subsequent monitoring.

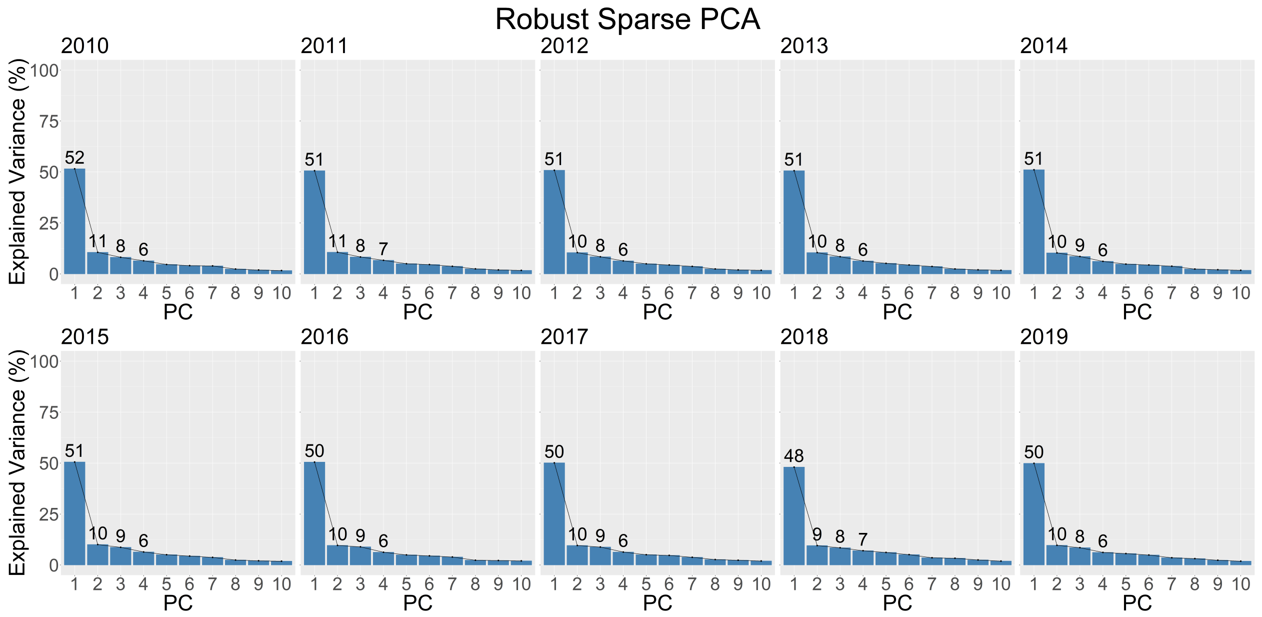

Supplementary Figure F5: Scree plot for Robust Sparse PCA method. Figure is generated with R software[1].

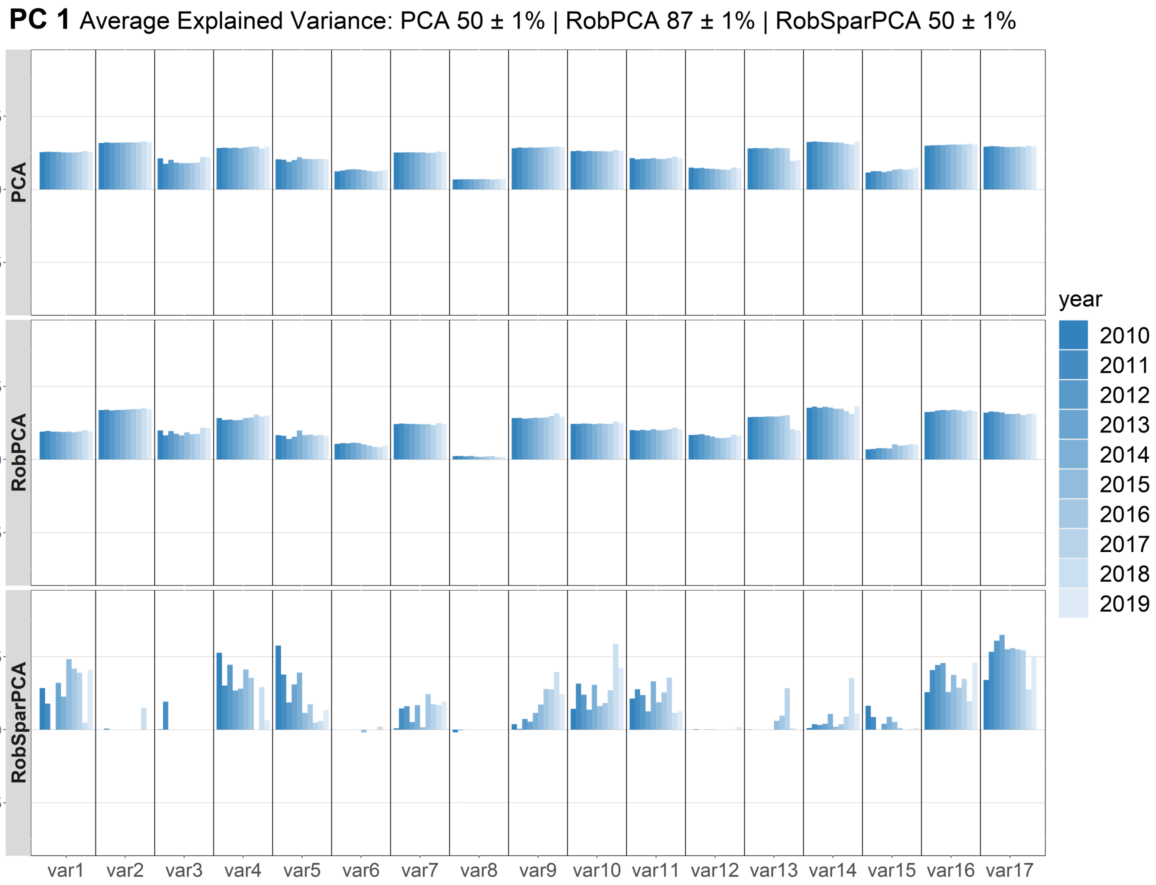

Supplementary Figure F6: Loading plot for all PCA methods. Figure is generated with R software[1].

4 Loadings Plot for DFM method

The loadings $\mathbf{C}^i$ for the i-th country are stacked into the diagonal matrix $\mathbf{C}$, whereas the cross-country interactions are introduced by the matrix $\hat{\boldsymbol{A}}$ estimated with VAR. Our setting force the $\mathbf{C}^i$ to be constant so we can estimate loadings for each country-variable pair. Therefore, for ease of visualization, figure F7 reports the distribution of the loadings for each input variable over the 168 countries, representing the average trend over the years. The bimodal shape of all distributions implies a clear discriminative power of the index between less risky countries and riskier ones.

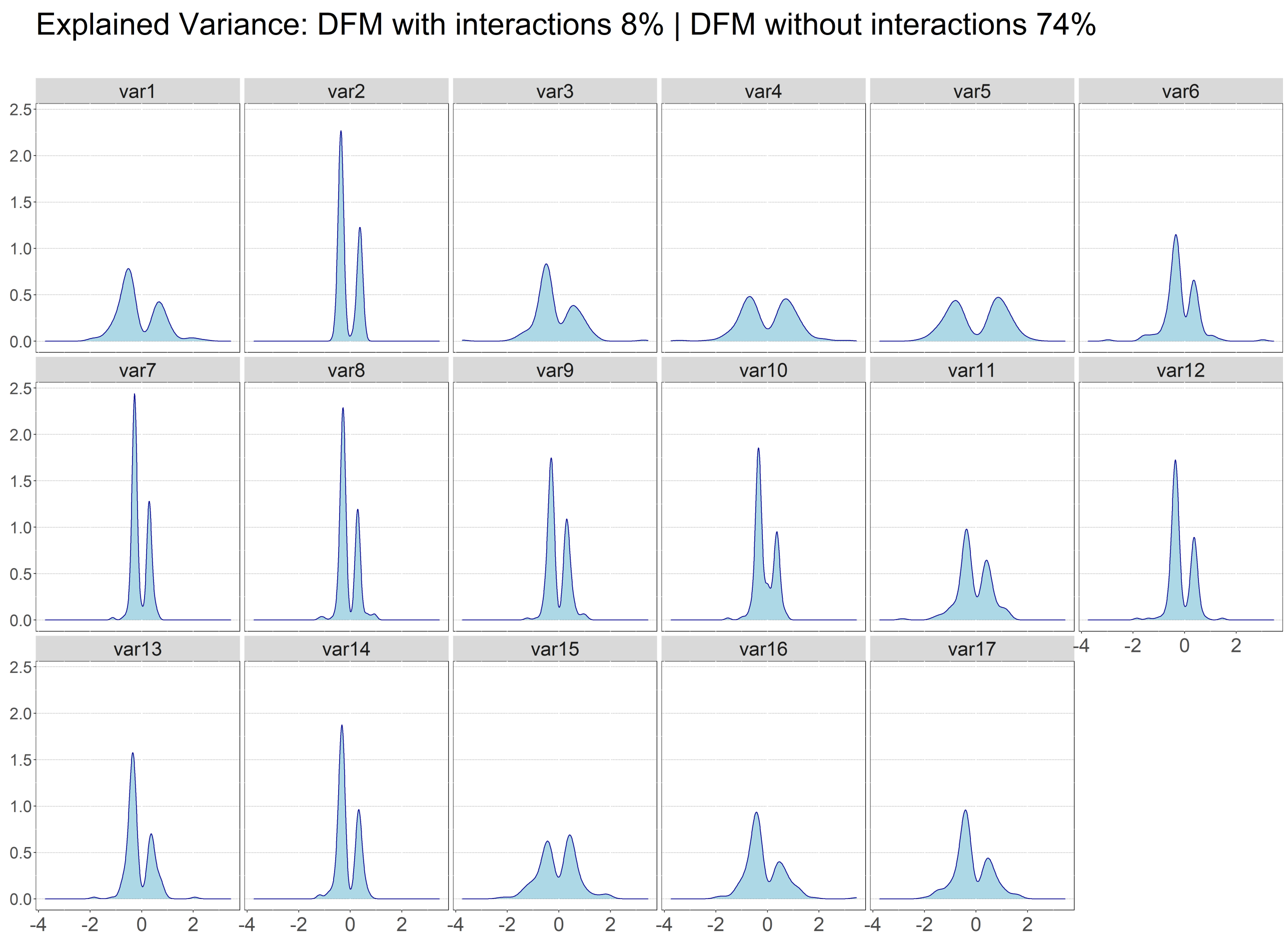

Supplementary Figure F7: Loading plot for DFM method. On x-axis is reported the logarithm of loading values. Figure is generated with R software[1].

4.1 Index Robustness Check

Our robustness check is performed by using the ESR index as an input variable in supervised regressions. The aim is to evaluate the fitting power of the summary index compared to the original variables in modeling some relevant macro economic indicators. From Figure F8 through Figure F13 we report percent increase of RMSE in predicting macro economic indicators of interest (Unenployment, Real GDP per capita, Share of government consumption, Price level of capital information, Trade Volume, Outstanding Loans of Commercial banks) due to the employment of the ESR index. The graphs report comparison between regressions with the single continuous ESR index as regressor and the one with original variables. In this way we assess how much the RMSE increases by substituting 17 variables with our summary index. In table T5 we report numerical results for the regressions above described.

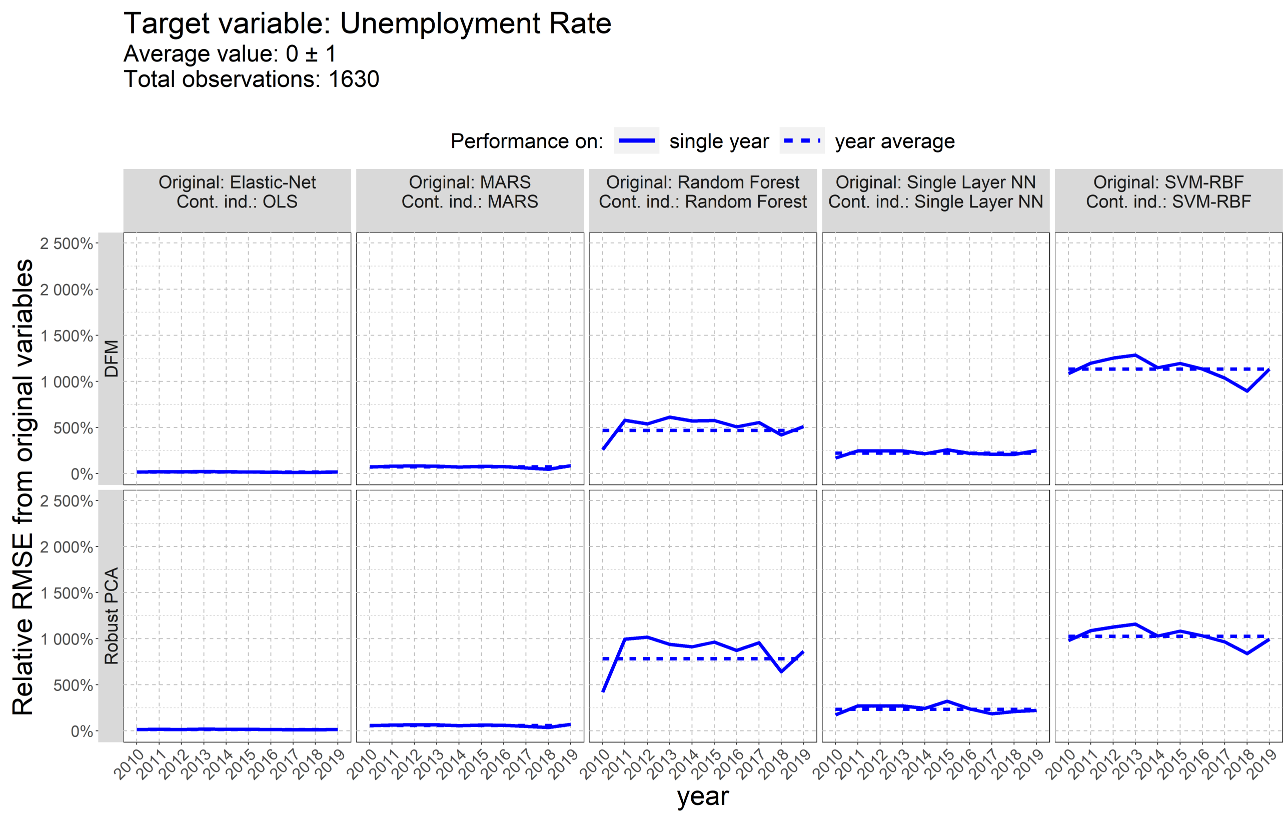

Supplementary Figure F8: RMSE percent increase in predicting Unemployment rate. Comparison between regression with the single continuous index as regressor and the one with original variables. Solid lines show the single year metrics, dashed lines show the full dataset, i.e. average over years, metric. Figure is generated with R software[1].

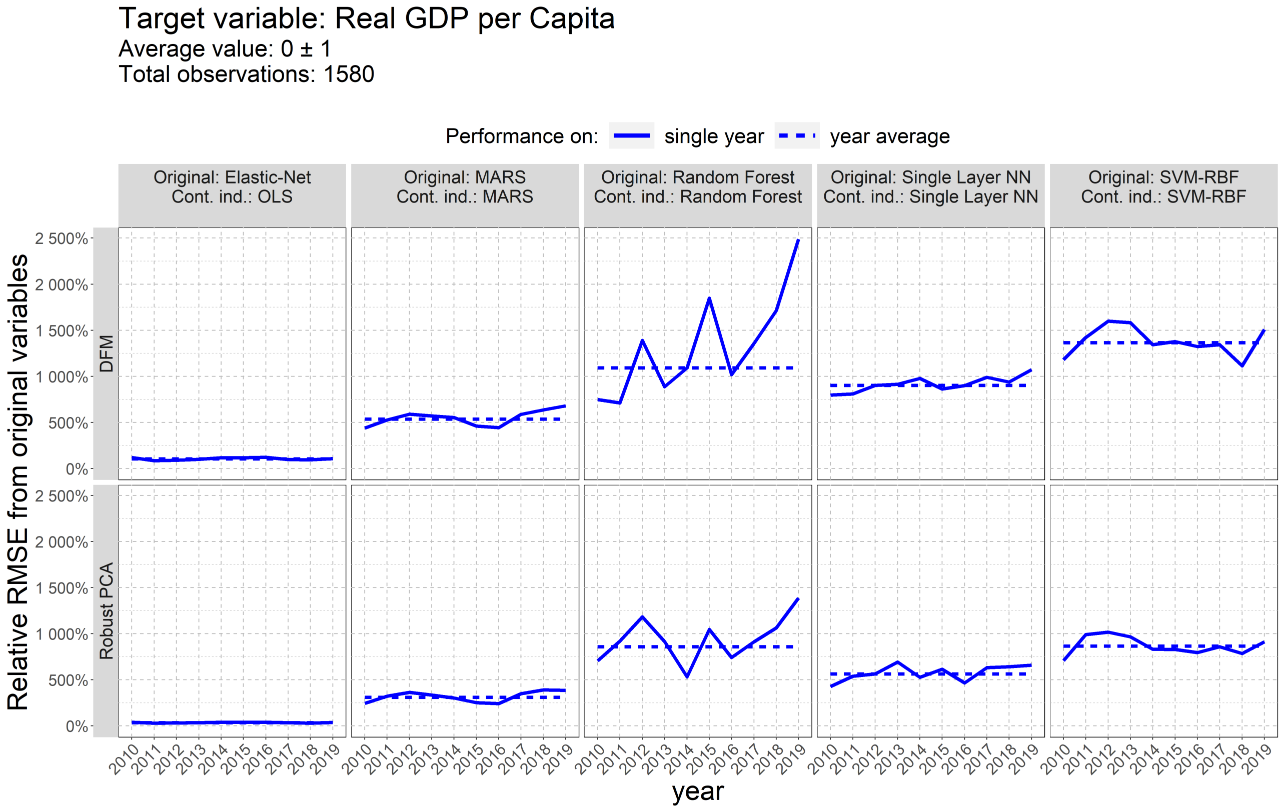

Supplementary Figure F9: RMSE percent increase in predicting Real GDP per capita. Comparison between regression with the single continuous index as regressor and the one with original variables. Solid lines show the single year metrics, dashed lines show the full dataset, i.e. average over years, metric. Figure is generated with R software[1].

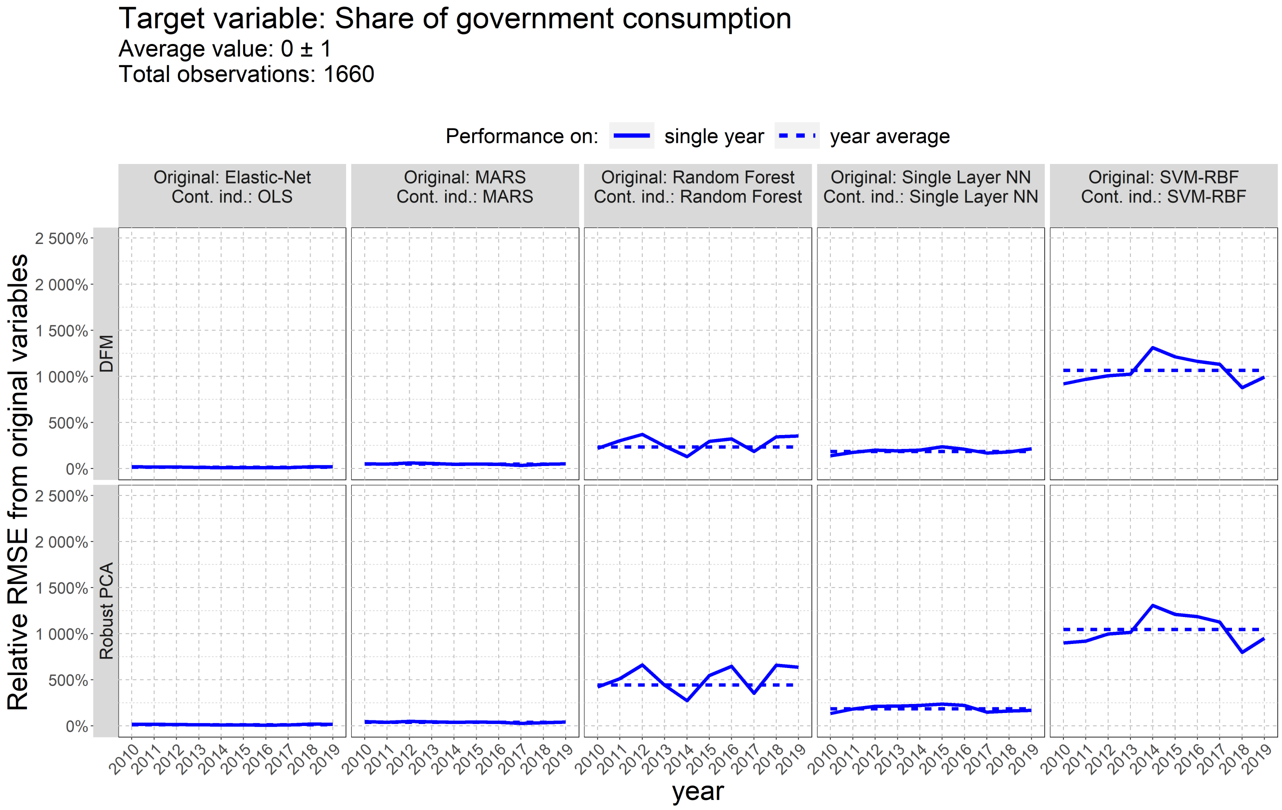

Supplementary Figure F10: RMSE percent increase in predicting Share of government consumption. Comparison between regression with the single continuous index as regressor and the one with original variables. Solid lines show the single year metrics, dashed lines show the full dataset, i.e. average over years, metric. Figure is generated with R software[1].

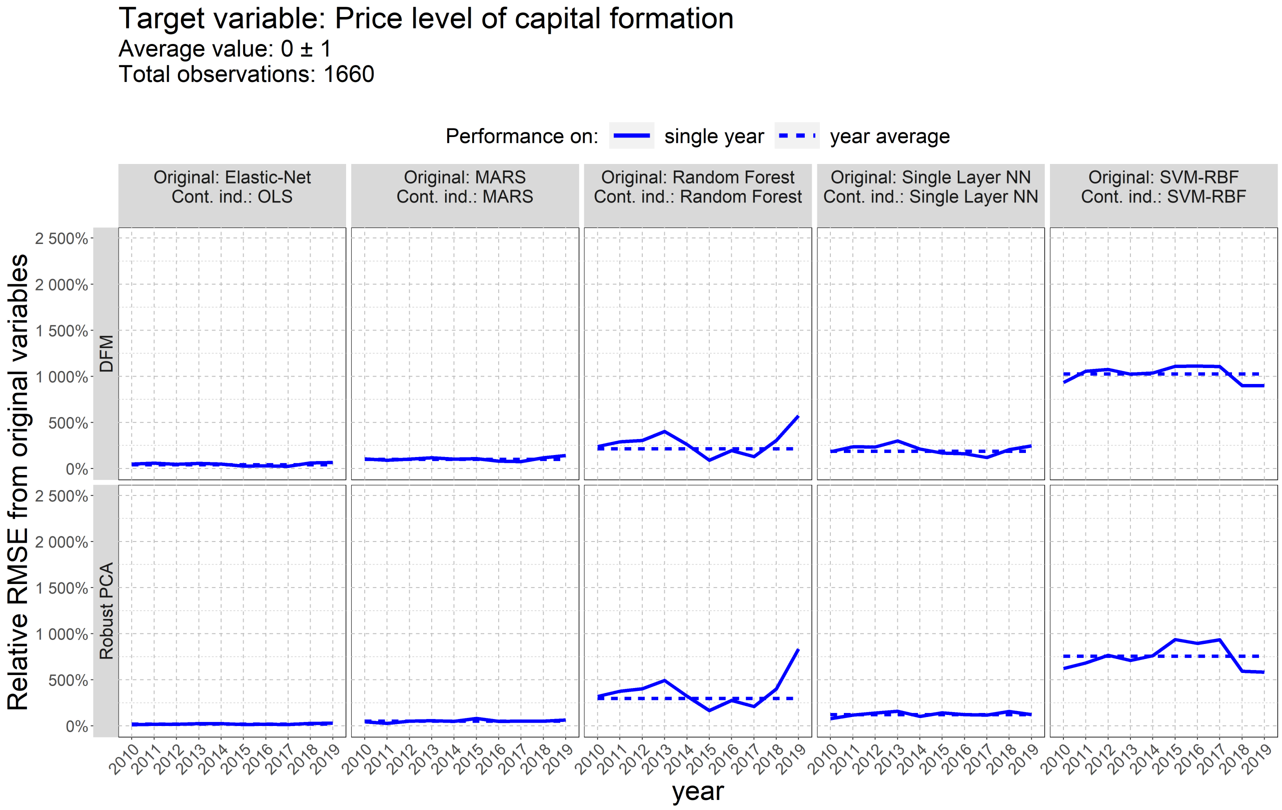

Supplementary Figure F11: RMSE percent increase in predicting Price level of capital formation. Comparison between regression with the single continuous index as regressor and the one with original variables. Solid lines show the single year metrics, dashed lines show the full dataset, i.e. average over years, metric. Figure is generated with R software[1].

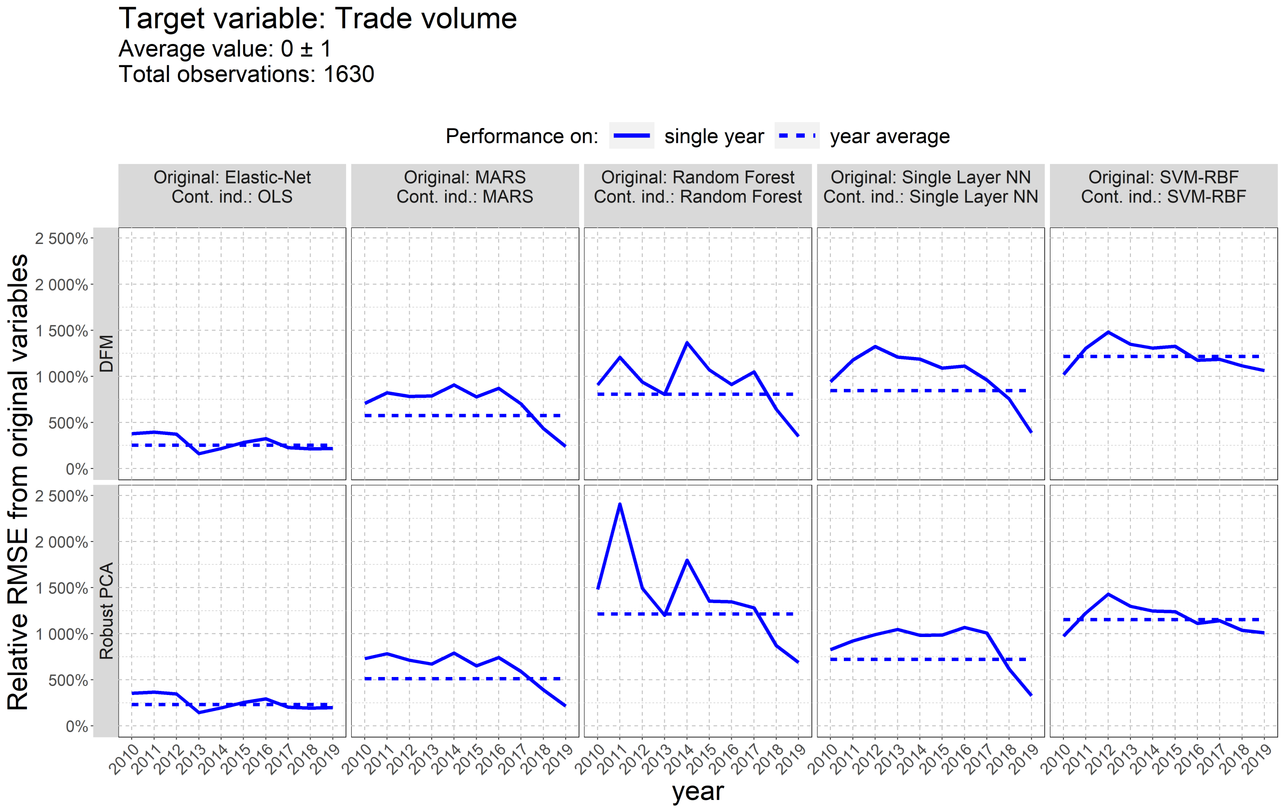

Supplementary Figure F12: RMSE percent increase in predicting Trade volume. Comparison between regression with the single continuous index as regressor and the one with original variables. Solid lines show the single year metrics, dashed lines show the full dataset, i.e. average over years, metric. Figure is generated with R software[1].

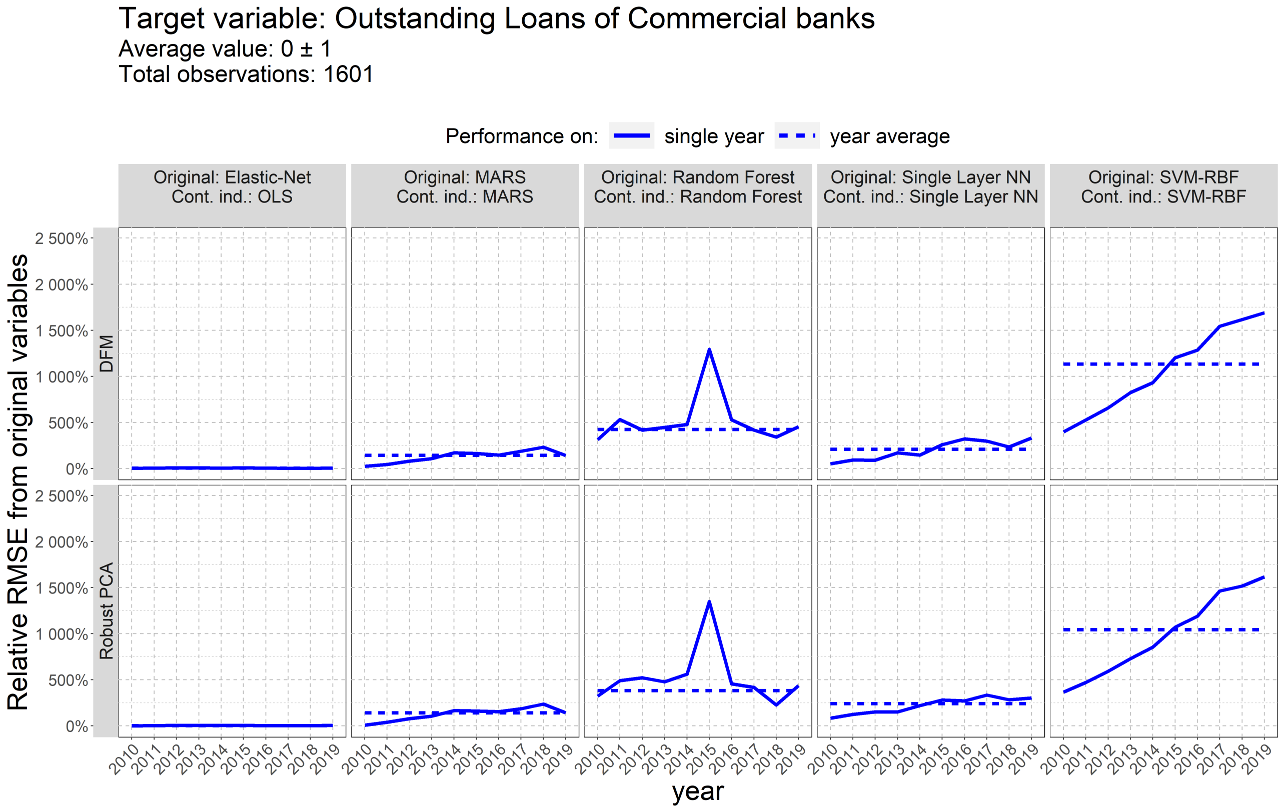

Supplementary Figure F13: RMSE percent increase in predicting Outstanding Loans of Commercial banks. Comparison between regression with the single continuous index as regressor and the one with original variables. Solid lines show the single year metrics, dashed lines show the full dataset, i.e. average over years, metric. Figure is generated with R software[1].

Supplementary Table T5: RMSE in predicting macro-economic variables with continuous index as regressor. RMSE for regression with original variables is reported in parenthesis.

	RMSE index (RMSE original)					
Target variable	Outstanding Loans of Commercial banks		Price level of capital formation		Real GDP per Capita	
Algorithm	DFM	Robust PCA	DFM	Robust PCA	DFM	Robust PCA
Elastic-Net	0.998(0.962)	1(0.962)	1(0.705)	0.839(0.705)	1(0.492)	0.663(0.492)
MARS	0.995(0.409)	0.986(0.409)	1(0.502)	0.764(0.502)	0.987(0.155)	0.634(0.155)
Random Forest	0.854(0.163)	0.914(0.163)	0.432(0.137)	0.549(0.137)	0.479(0.04)	0.395(0.04)
SVM-RBF	1.001(0.081)	1(0.081)	1.005(0.089)	0.764(0.089)	1.027(0.07)	0.664(0.07)
Single Layer NN	0.991(0.321)	0.976(0.321)	0.994(0.347)	0.768(0.347)	0.997(0.099)	0.663(0.099)

Target variable	Share of government consumption		Trade volume	
Algorithm	DFM	Robust PCA	DFM	Robust PCA
Elastic-Net	1(0.887)	0.999(0.887)	1(0.283)	0.935(0.283)
MARS	1(0.679)	0.948(0.679)	1(0.148)	0.909(0.148)
Random Forest	0.445(0.133)	0.719(0.133)	0.437(0.048)	0.637(0.048)
SVM-RBF	0.992(0.085)	0.977(0.085)	1.011(0.077)	0.954(0.077)
Single Layer NN	0.994(0.35)	0.973(0.35)	0.995(0.105)	0.926(0.105)

4.2 Index evolution over years

From F14 through F17 we report the evolution across time of the ESR index based on the two competing techniques for the different considered countries. It clearly emerges the higher sensitivity of the ESR index based on the DFM approach to the temporal dynamics which are explicitly modelled. PCA instead produces a rather flat pattern in line with the no direct modelling of the available years.

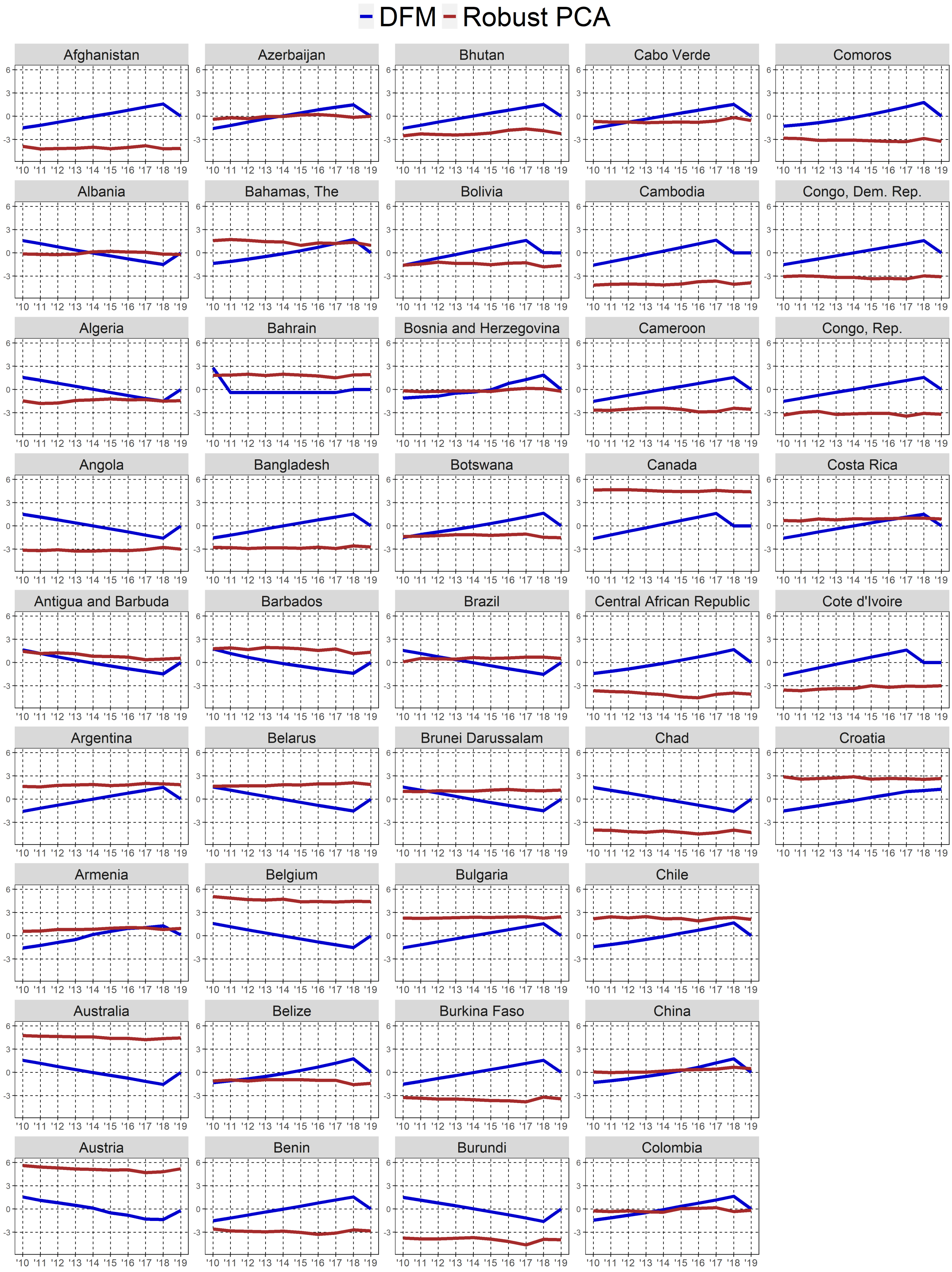

Supplementary Figure F14: Index evolution over years. Figure is generated with R software[1].

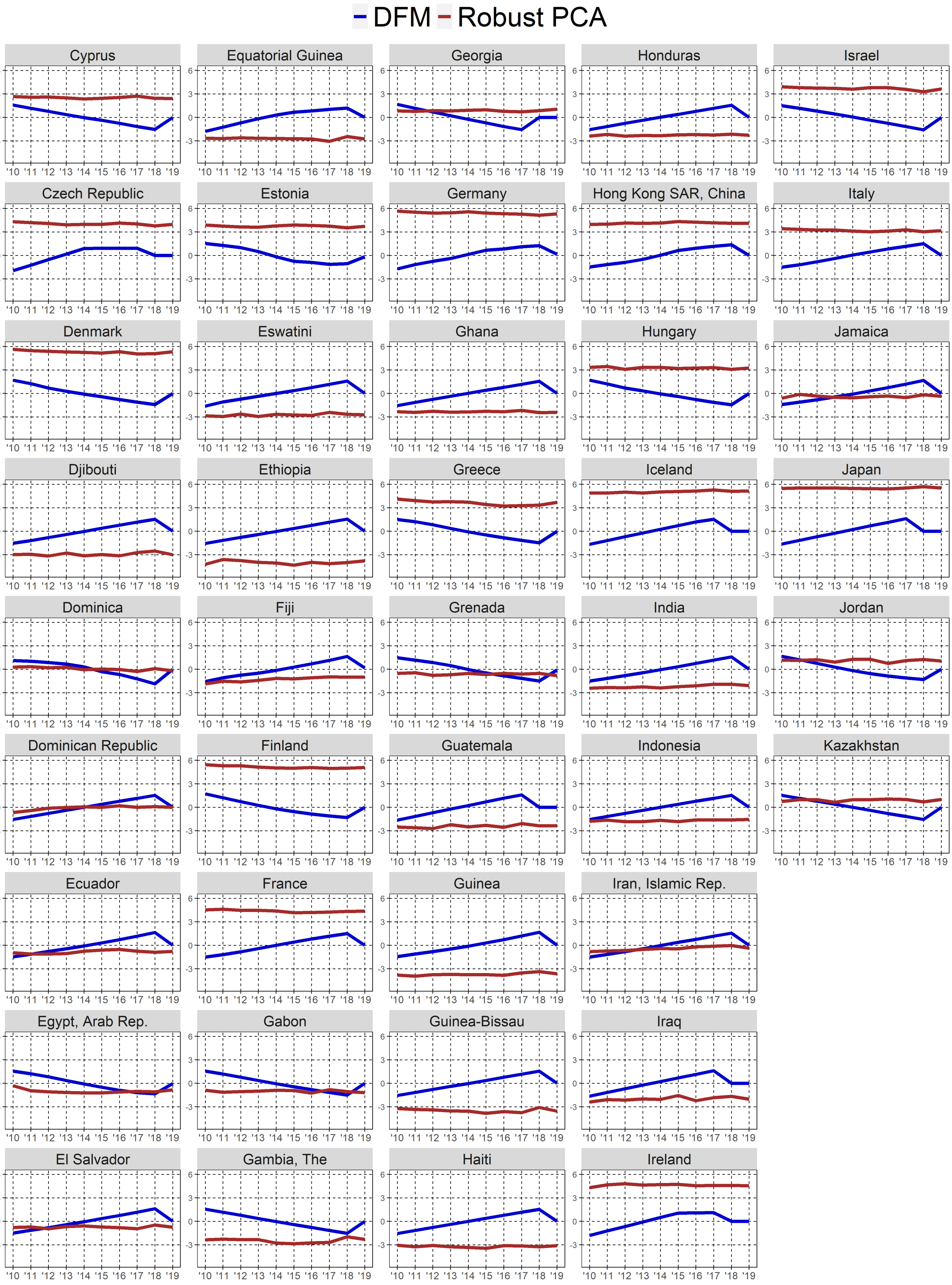

Supplementary Figure F15: Index evolution over years. Figure is generated with R software[1].

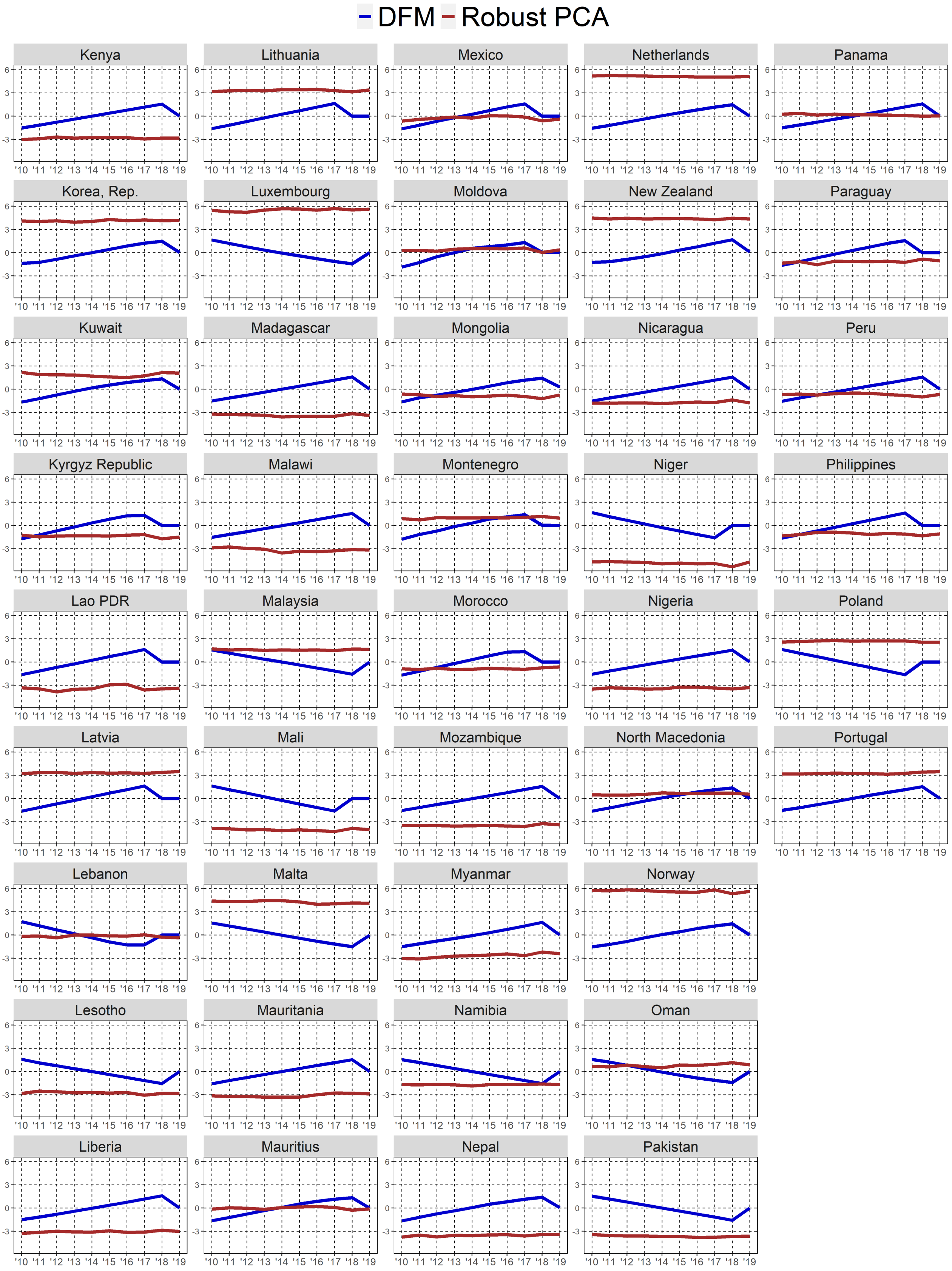

Supplementary Figure F16: Index evolution over years. Figure is generated with R software[1].

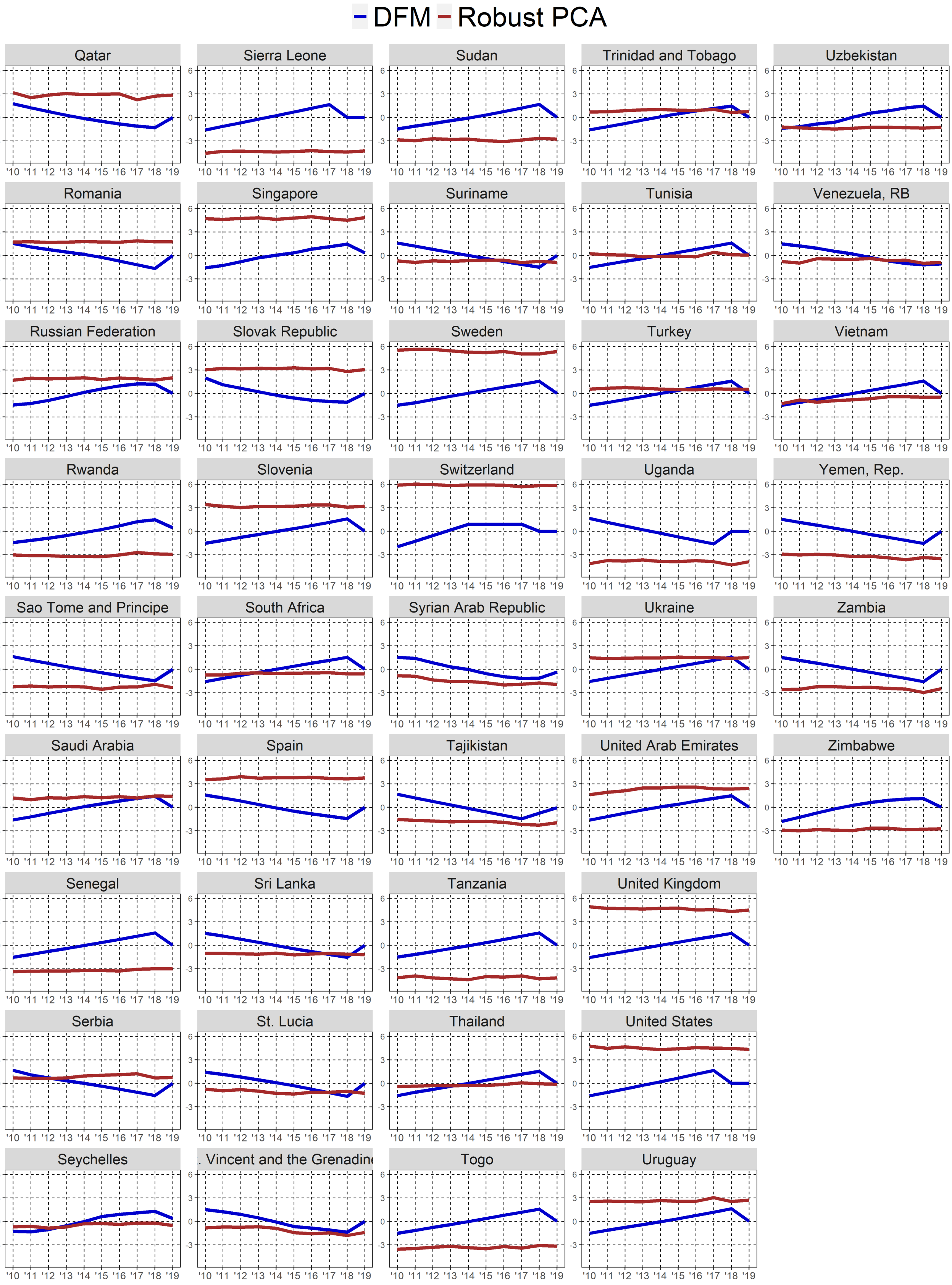

Supplementary Figure F17: Index evolution over years. Figure is generated with R software[1].

Supplementary References

[1] R Core Team. *R: A Language and Environment for Statistical Computing*. R Foundation for Statistical Computing, Vienna, Austria, 2021.

[2] D. R. Johnson and R. Young. Toward best practices in analyzing datasets with missing data, comparisons and recommendations. *Journal of Marriage and Family*, 73:926–45, 2011.

[3] G. King, J. Honaker, A. Joseph, and K. Scheve. Analyzing incomplete political science data: An alternative algorithm for multiple imputation. *American Political Science Review*, 95:49–69, 2001.

[4] T. Hastie, R. Mazumder, J. D. Lee, and R. Zadeh. *Matrix completion and low-rank SVD via fast alternating least squares*. 2015.

[5] S. A. Khan and M. Ammad-ud-din. *tensorBF: an R package for Bayesian tensor https://www.biorxiv.org/content/biorxiv/early/2016/12/29/097048.full.pdf*. 2016.

[6] M. Nardo, M. Saisana, A. Saltelli, S. Tarantola, A. Hoffman, and E. Giovannini. Handbook on constructing composite indicators: methodology and user guide. *OECD Statistics Working Paper STD/DOC*, 2005.
